# Effect of Financial Bonus Size, Loss Aversion, and Increased Social Pressure on Physician Pay-for-Performance: A Randomized Clinical Trial and Cohort Study

**DOI:** 10.1001/jamanetworkopen.2018.7950

**Published:** 2019-02-08

**Authors:** Amol S. Navathe, Kevin G. Volpp, Kristen L. Caldarella, Amelia Bond, Andrea B. Troxel, Jingsan Zhu, Shireen Matloubieh, Zoe Lyon, Akriti Mishra, Lee Sacks, Carrie Nelson, Pankaj Patel, Judy Shea, Don Calcagno, Salvatore Vittore, Kara Sokol, Kevin Weng, Nichia McDowald, Paul Crawford, Dylan Small, Ezekiel J. Emanuel

**Affiliations:** 1 Center for Health Equity Research and Promotion, Corporal Michael J. Crescenz Veterans Affairs Medical Center, Philadelphia, Pennsylvania; 2 Department of Medical Ethics and Health Policy, Perelman School of Medicine, University of Pennsylvania, Philadelphia; 3 Center for Health Incentives and Behavioral Economics, University of Pennsylvania, Philadelphia; 4 Department of Healthcare Policy and Research, Weill Cornell Medicine, New York, New York; 5 Department of Health Care Management, Wharton School of Business, University of Pennsylvania, Philadelphia; 6 Department of Population Health, School of Medicine, New York University, New York, New York; 7 Advocate Physician Partners, Downers Grove, Illinois; 8 Division of General Internal Medicine, Perelman School of Medicine, University of Pennsylvania, Philadelphia

## Abstract

**Importance:**

Despite limited effectiveness of pay-for-performance (P4P), payers continue to expand P4P nationally.

**Objective:**

To test whether increasing bonus size or adding the behavioral economic principles of increased social pressure (ISP) or loss aversion (LA) improves the effectiveness of P4P.

**Design, Setting, and Participants:**

Parallel studies conducted from January 1 to December 31, 2016, consisted of a randomized clinical trial with patients cluster-randomized by practice site to an active control group (larger bonus size [LBS] only) or to groups with 1 of 2 behavioral economic interventions added and a cohort study comparing changes in outcomes among patients of physicians receiving an LBS with outcomes in propensity-matched physicians not receiving an LBS. A total of 8118 patients attributed to 66 physicians with 1 of 5 chronic conditions were treated at Advocate HealthCare, an integrated health system in Illinois. Data were analyzed using intention to treat and multiple imputation from February 1, 2017, through May 31, 2018.

**Interventions:**

Physician participants received an LBS increased by a mean of $3355 per physician (LBS-only group); prefunded incentives to elicit LA and an LBS; or increasing proportion of a P4P bonus determined by group performance from 30% to 50% (ISP) and an LBS.

**Main Outcomes and Measures:**

The proportion of 20 evidence-based quality measures achieved at the patient level.

**Results:**

A total of 86 physicians were eligible for the randomized trial. Of these, 32 were excluded because they did not have unique attributed patients. Fifty-four physicians were randomly assigned to 1 of 3 groups, and 33 physicians (54.5% male; mean [SD] age, 57 [10] years) and 3747 patients (63.6% female; mean [SD] age, 64 [18] years) were included in the final analysis. Nine physicians and 864 patients were randomized to the LBS-only group, 13 physicians and 1496 patients to the LBS plus ISP group, and 11 physicians and 1387 patients to the LBS plus LA group. Physician characteristics did not differ significantly by arm, such as mean (SD) physician age ranging from 56 (9) to 59 (9) years, and sex (6 [46.2%] to 6 [66.7%] male). No differences were found between the LBS-only and the intervention groups (adjusted odds ratio [aOR] for LBS plus LA vs LBS-only, 0.86 [95% CI, 0.65-1.15; *P* = .31]; aOR for LBS plus ISP vs LBS-only, 0.95 [95% CI, 0.64-1.42; *P* = .81]; and aOR for LBS plus ISP vs LBS plus LA, 1.10 [95% CI, 0.75-1.61; *P* = .62]). Increased bonus size was associated with a greater increase in evidence-based care relative to the comparison group (risk-standardized absolute difference-in-differences, 3.2 percentage points; 95% CI, 1.9-4.5 percentage points; *P* < .001).

**Conclusions and Relevance:**

Increased bonus size was associated with significantly improved quality of care relative to a comparison group. Adding ISP and opportunities for LA did not improve quality.

**Trial Registration:**

ClinicalTrials.gov Identifier: NCT02634879

## Key Points

**Question:**

Does increasing bonus size or adding the behavioral economic principles of social pressure or loss aversion improve pay-for-performance effectiveness among physicians?

**Findings:**

In this randomized clinical trial of 54 physicians and cohort study including 66 physicians and 8188 patients, increased bonus size was associated with improved quality relative to a comparison group, although adding increased social pressure and opportunities for loss aversion did not improve quality.

**Meaning:**

Increasing pay-for-performance bonus sizes may be associated with improved effectiveness, whereas adding the behavioral economic principles of social pressure and loss aversion may not be.

## Teaser

This randomized clinical trial and cohort study tests whether increasing bonus size or adding the behavioral economic principles of social pressure or loss aversion improves the effectiveness of pay-for-performance among a sample of physicians and their patients.

## Introduction

Pay-for-performance (P4P) is being increasingly used by health insurers and health care systems to incentivize physicians to practice higher-value medicine. The introduction of the Merit Incentive Payment System as part of the Medicare Access and CHIP (Children's Health Insurance Program) Reauthorization Act has made P4P a centerpiece of the US shift from volume to value.^1,2,3^

However, P4P has not produced consistently positive results.^2,4,5,6,7,8^ Several explanations have been proposed, including that incentive sizes are too small and baseline performance on quality metrics is too high, reflecting little opportunity to improve; extrinsic incentives may crowd out intrinsic motivation; and the design of programs (eg, high-performance targets) only incentivizes physicians with performance near the thresholds.^2,4,5,6,7,8^ Further, few randomized clinical trials (RCTs) have evaluated traditional financial P4P incentives for physicians in pragmatic settings, that is, across payers and with incentives tied to comprehensive sets of quality metrics.^9,10^ Systematic reviews have suggested that programs with larger bonuses achieve greater effects, but they have not accounted for potential confounding factors.^6,7,8^ Whether increasing P4P bonus sizes is effective remains an open question.

Another promising strategy is to apply principles from behavioral economics to the design of P4P incentives to make them more effective within the same budget.^11,12,13^ Although behavioral economics principles have been applied extensively to financial incentives for patients, few have been rigorously applied to financial incentives for physicians.^13,14,15^

To address these knowledge gaps, we conducted a pragmatic RCT of P4P and a simultaneous prospective quasi-experimental comparison evaluating the following 2 interventions: (1) the addition of the behavioral economic principles of loss aversion (LA) and increased social pressure (ISP) to larger bonus sizes (LBS) and (2) LBS alone.

## Methods

### Study Design

This study follows the Consolidated Standards of Reporting Trials (CONSORT) reporting guideline and the Strengthening the Reporting of Observational Studies in Epidemiology (STROBE) reporting guideline. Details of the study design, randomization scheme, and interventions are summarized below and presented in detail in the original protocol (Supplement 1). The study protocol was approved by the institutional review boards at the University of Pennsylvania (Philadelphia) and Advocate Health Care (Downers Grove, Illinois), including a waiver of informed consent for patients and physicians.

### Setting

The study was conducted in a clinically integrated physician network led by Advocate Physicians Partners (Advocate), Downers Grove, Illinois. The Trinity physician-hospital organization was the setting for the RCT, with other Advocate practices (non-Trinity) serving as the comparison group for the cohort study. Compared with non-Trinity practices, Trinity serves a patient population that includes a higher proportion of minority patients as well as lower socioeconomic status communities. Trinity was selected for inclusion in the RCT by Advocate leadership because its physicians’ quality scores had been consistently lower than those of non-Trinity physicians, all of whom participate in the same P4P program.

This pragmatic study was thus part of a quality improvement study led by Advocate leadership. Budgetary limitations prevented testing LBS across the network of more than 4000 physicians, and it was not possible to randomize to LBS vs behavioral economic designs because it was not culturally acceptable to give different sized bonuses to physicians achieving identical quality scores. Hence, we prospectively designed the study as an observational analysis of LBS by applying them to all Trinity physicians and then randomizing them to evaluate the effect of adding LA and ISP. However, patient-level data became available after randomization that demonstrated fewer physicians with uniquely attributed patients (ie, in our design a patient could only be attributed to 1 physician) and thus led to a smaller sample size than at randomization.

The interventions were tested in a P4P program that started in 2005 with large incentives, for which the design has been reported previously.^16^ Briefly, the P4P program used a composite quality score (the clinical integration score), which represents a weighted percentage of evidence-based quality measures achieved. The P4P bonus paid is in proportion to the clinical integration score percentage with the maximum possible bonus determined by panel size and other factors such as payer mix.

Advocate leadership and the study investigators conducted in-person sessions with all eligible physicians to describe the study procedures and conduct a baseline survey. All physicians in the Trinity leadership voted to participate in the study. All Advocate-wide physicians were aware of the study because of networkwide communications and were routinely monitored per the existing P4P program.

### Study Patient Population

The study patient population included patients with at least 1 of 5 chronic diseases (asthma, chronic obstructive pulmonary disease, type 2 diabetes, coronary artery disease or ischemic vascular disease, and congestive heart failure). Participating patients were attributed (using plurality of visits) for more than 12 months to participating physicians who were active in Advocate’s existing P4P program.

### Interventions

The active phase of the trial was from January 1 through December 31, 2016. The LBS intervention provided maximum P4P bonuses larger than previous years by a mean of $3355 per physician, representing an approximately 32% increase in bonus size and an increase of $16 per patient (from $52 to $68 per patient). Quality metrics and scoring methods were left unchanged. This intervention represented an active control condition in which the physicians received larger bonuses than physicians not participating in the RCT (and larger bonuses than they received the prior year).

The LBS plus LA intervention included the larger maximum bonus plus prefunded incentives in a virtual health system bank account in the physician’s name. The prefunded incentives, which were 50% of the expected incentives based on prior year’s performance, were placed into the virtual account on January 1, 2016, and accessible by email request to the Advocate chief financial officer. If at the end of 2016 physicians earned less P4P dollars than were placed in the virtual accounts, physicians were required to pay back funds from the virtual account and/or any dollars overdrawn from it. Physicians in this intervention group received 4 additional pro formas in February, July, September, and November of 2016 that indicated dollar amounts for the prefunded incentive, those accessed year-to-date, the projected 2016 incentive bonus size, and the projected residual unearned incentive (eFigure 1 in Supplement 2). This intervention group was exposed to the behavioral economic principles of an endowment effect, in which people work harder not to give up something they already have, and LA, testing whether physicians would respond more strongly to the same incentive amount framed in terms of losses rather than gains. However, the risk of overdrawing was quite small, because 94% of physicians earned at least 50% of their bonuses in the prior year, meaning the intervention may better be interpreted as the opportunity for LA.

The LBS plus ISP intervention included the increased maximum bonus but also changed the composite quality score from 70% based on individual score and 30% based on the physician-hospital organization score to 50% individual and 50% group (herein defined as all physicians in the same intervention group). Physicians in this intervention group also received 4 additional pro formas on the same dates as above with the additional P4P bonus dollars that would be earned by the 20–percentage point increase in the weighting given to group score as well as an unblinded list of physicians with performance scores on 2 of the quality measures (eFigures 1 and 2 in Supplement 2). Physicians with scores below the performance threshold were identified. This intervention used the behavioral economic principle of ISP, through which individuals will try to improve their performance because others in the group can identify poor performers.

### Outcome Measures

The primary study outcome was the 2015-2016 change in proportion of applicable chronic disease and preventive evidence-based measures within the P4P program meeting or exceeding benchmarks based on national Healthcare Effectiveness Data and Information Set standards at the patient level, representing a patient’s view of the proportion of evidence-based care received. Secondary outcomes included changes in the probabilities of meeting the individual measures (eTable 1 in Supplement 2).

### Randomization

Eligible affiliated physicians in the RCT were randomized by practice site to active control or 1 of 2 intervention groups in a 1:1:1 ratio, stratified by primary care vs specialist. Because of the need to randomize before the start of 2016 (because quality metrics were on a calendar-year cycle) and owing to data transfer delays, randomization occurred before data for attribution of patients became available. Study participants and operational staff did not have any influence on randomization but were not blinded to group assignment because knowledge of the incentives is essential to their mechanism. Study investigators and data analysts remained blinded until all follow-up data were obtained and primary analyses were finalized.

### Statistical Analysis

Data were analyzed using intention to treat and multiple imputation. Data analysis was performed from February 1, 2017, through May 31, 2018.

#### RCT Testing Addition of ISP and LA to LBS

Although randomization occurred at the physician-site level, the patient was the unit of analysis for the primary outcome. The patient-level primary outcome was measured using the number of measures achieved (events) of all applicable chronic disease measures (trials). The primary analysis used a generalized linear model with binomial distribution and logit link function to estimate the odds of achieving evidence-based chronic disease measures for each patient (ie, events and/or trials) clustered at the physician level to adjust for multiple patients nested within physicians.^16,17,18^ The model included adjustments for the overall proportion of measures achieved in 2016, each treatment group, and the interaction term for treatment group by 2016, which gave the primary effect of interest representing the change in the outcome for each treatment group compared with the change in the active control group. Examining the change was intended to address potential differences in baseline measures between groups. Additional adjustments included patient demographics (including race) and chronic condition and physician demographics, training and specialty, certification, years of experience, and practice characteristics. We conducted pairwise comparisons of the primary outcome for each treatment group against the control group and for the treatment groups compared with each other, exponentiating the coefficient and 95% CI estimates from the logistic regressions to compute adjusted odds ratios (aORs).

Approximately 11% of patients were missing follow-up quality measures included in the primary outcome. Multiple imputation with 20 imputations was used, achieving at least 98% relative efficiency and ensuring in-range values. All analyses were conducted on each imputed data set; results were combined using the standard rules from Rubin.^18^ We also conducted sensitivity analysis without imputing missing data, clustering by site (although most sites only had 1 physician), and with models using physician random effects.

Power calculations were derived assuming the comparison of each incentive group with the control group using a Bonferroni-corrected type I error of 0.017, followed by comparison of any incentive groups that showed significant differences from control using a sequential Holm-Bonferroni approach.^19^ The study was designed to have at least 80% power to detect differences in the change in proportion of evidence-based measures received between any incentive group and control of 5%. We used a conservative assumption of intraclass correlation coefficient of 0.25 within physicians. Simulation studies incorporating these variables indicated the need for approximately 3420 participants (1140 per group).

#### Cohort Study Testing LBS

To examine the association between the mean effect across all 3 groups receiving the LBS intervention and the proportion of evidence-based care received by patients, we used a difference-in-differences method to compute the change in the primary outcome for patients attributed to physicians in the LBS group vs those attributed to a propensity-matched set of physicians who did not receive an LBS.^20^ Physicians were matched using demographics, the preintervention (2015) level, and the preintervention time trend in the primary outcome (eMethods 1, eFigure 3, and eFigure 4 in Supplement 2).^21^ The time trend was measured as the difference between 2014 and 2015 risk-adjusted composite scores. As in the RCT analysis and other literature,^22^ we used the same primary outcome and specification, with the addition of physician fixed effects to account for unobserved time-invariant differences among physicians because of a lack of randomization. Model variables included the main intervention group effect, year effect, and interaction of group and year for the effect of interest. A test of trends between the groups from 2011 to 2015 did not indicate divergent trends (eMethods 2 and eTable 5 in Supplement 2). Standard errors were clustered to account for repeated measures at the patient level following previous studies.^16,23^ We also conducted a sensitivity analysis without physician fixed effects.

#### Estimated Risk-Standardized Primary Outcome

In the RCT and cohort study analyses, we estimated the risk-standardized proportion of evidence-based measures achieved using bootstrapping.^22,24^ All *P* values were 2-sided with *P* < .05 indicating significance. Analyses were conducted using SAS software (version 9.4; SAS Institute, Inc).

#### Physician Survey Methods

We also conducted online pretrial and posttrial physician surveys to assess the influence and acceptability of the interventions in several domains using paired *t* tests to compare mean Likert scale responses by group. The domains included baseline attitudes, teamwork, financial salience, practice environment, awareness and/or understanding, influence on clinical behavior, and unintended consequences.

## Results

### Sample Characteristics

**Figure 1.**
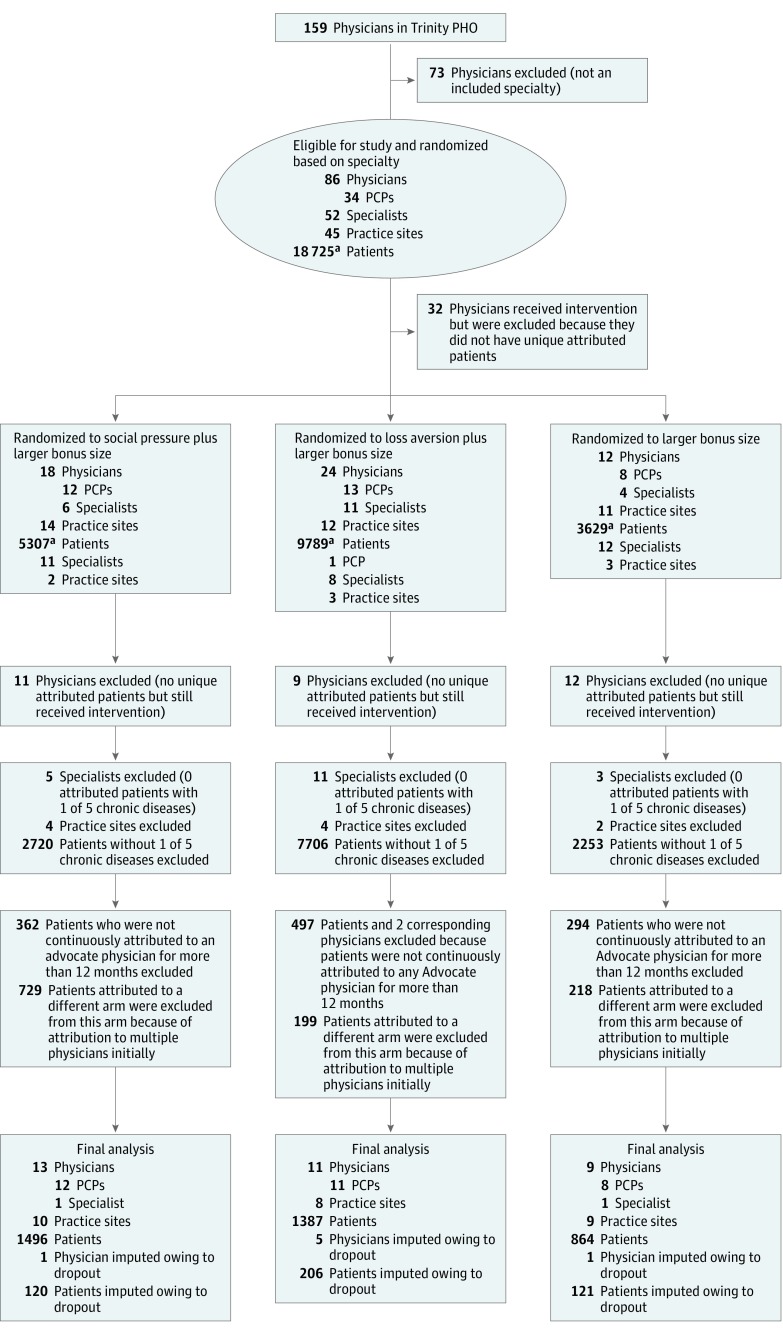
Flow Diagram of Physician, Patient, and Site Progress Through the Trial LBS indicates larger bonus size; PCP, primary care physician; and PHO, physician-hospital organization. ^a^Patients were not uniquely attributed to 1 physician at this stage. The total number of unique patients was 16 815.

A total of 86 physicians were randomized, although 32 received the interventions but were excluded from the analysis because they did not have unique attributed patients (Figure 1). Seven physicians (with 465 attributed patients) electively terminated their contracts with Advocate for reasons outside of the study, but patients were analyzed according to the assigned study group in an intention-to-treat approach. A total of 33 physicians (18 male [54.5%] and 15 female [45.5%]), 27 practice sites, and 3747 attributed patients (1358 male [36.2%] and 2384 female [63.6%] among those with available data; mean [SD] age, 64 [18] years) were included in the final RCT analysis. Nine physicians and 864 patients were randomized to the LBS-only group, 13 physicians and 1496 patients to the LBS plus ISP group, and 11 physicians and 1387 patients to the LBS plus LA group. Physician characteristics did not differ significantly by arm, such as mean (SD) physician age ranging from 56 (9) to 59 (9) years, and sex (6 [46.2%] to 6 [66.7%] male). Mean (SD) physician age was 57 (10) years, with a mean (SD) tenure of 12 (8) years with Advocate and predominantly in a primary care specialty (27 of 33 [81.8%]) (Table 1). Demographic and professional characteristics of enrolled physicians and attributed patients were not significantly different across intervention groups. There were small differences in patient race and age. Characteristics of the 33 matched physicians did not exhibit differences relative to the RCT physicians (Table 2 and eTables 2 and 4 in Supplement 2), whereas their 4371 attributed patients were less likely to be black (831 of 4371 [19.0%] vs 2667 of 3747 [71.2%]; *P* < .001) and were older (median age, 67 years [interquartile range, 57-75 years] vs 64 years [interquartile range, 55-73 years]; *P* < .001) compared with the RCT patients.

**Table 1.** Physician and Patient Characteristics^a^

Characteristic	RCT Study Group				Cohort Study Group		
	LBS Plus ISP	LBS Plus LA	LBS Only	*P* Value	LBS	Non-LBS	*P* Value
**Physicians**							
No. of physicians	13	11	9	NA	33	33	NA
Age, mean (SD), y	56 (9)	56 (11)	59 (9)	.67	57 (10)	55 (8)	.27
Tenure, mean (SD), y	10 (8)	11 (8)	17 (4)	.07	12 (8)	12 (8)	.98
No. of patients, median (IQR)	91 (19-194)	27 (15-243)	80 (63-146)	.84	67 (19-157)	135 (28-189)	.36
Sex, No. (%)							
Male	6 (46.2)	6 (54.5)	6 (66.7)	.62	18 (54.5)	20 (60.6)	.62
Female	7 (53.8)	5 (45.4)	3 (33.3)		15 (45.4)	13 (39.4)	
Specialty, No. (%)^a^							
Family medicine	7 (53.8)	3 (27.3)	4 (44.4)	.54	14 (42.4)	15 (45.4)	>.99
Internal medicine	3 (23.1)	7 (63.6)	3 (33.3)		13 (39.4)	12 (36.4)	
Pediatrics	2 (15.4)	1 (9.1)	1 (11.1)		4 (12.1)	3 (9.1)	
Other	1 (7.7)^b^	0	1 (11.1)^c^		2 (6.1)^d^	3 (9.1)	
No. of chronic diseases treated, mean (SD)	1.61 (0.34)	1.61 (0.29)	1.56 (0.44)	.72	1.60 (0.34)	1.57 (0.29)	.65
**Patients**							
No. of patients	1496	1387	864	NA	3747	4371	NA
Age, median (IQR), y	62 (53-71)	66 (57-76)	65 (55-74)	<.001	64 (55-73)	67 (57-75)	<.001
Sex, No. (%)							
Male	495 (33.1)	529 (38.1)	334 (38.6)	.01	1358 (36.2)	2155 (49.3)	<.001
Female	997 (66.6)	857 (61.8)	530 (61.3)		2384 (63.6)	2203 (50.4)	
Unknown	4 (0.3)	1 (0.1)	0		5 (0.1)	13 (0.3)	
Race, No. (%)							
Black or African American	1213 (81.1)	875 (63.1)	579 (67.0)	<.001	2667 (71.2)	831 (19.0)	<.001
White	52 (3.5)	235 (16.9)	81 (9.4)		368 (9.8)	2666 (61.0)	
Other	29 (1.9)	61 (4.4)	59 (6.8)		149 (4.0)	313 (7.2)	
Unknown	202 (13.5)	216 (15.6)	145 (16.8)		563 (15.0)	561 (12.8)	
No. of chronic diseases, mean (SD)	1.64 (0.85)	1.64 (0.82)	1.49 (0.75)	<.001	1.6 (0.82)	1.65 (0.86)	.04
Patients in each chronic disease registry, No. (%)							
Asthma care	92 (6.1)	46 (3.3)	55 (6.4)	<.001	193 (5.2)	165 (3.8)	<.001
CHF	117 (7.8)	119 (8.6)	48 (5.6)	.03	284 (7.6)	333 (7.6)	.95
Controlling high blood pressure	1167 (78.0)	1190 (85.8)	579 (67.0)	<.001	2936 (78.4)	3522 (80.6)	.01
COPD	239 (16.0)	200 (14.4)	248 (28.7)	<.001	687 (18.3)	747 (17.1)	.14
Diabetes	587 (39.2)	416 (30.0)	231 (26.7)	<.001	1234 (32.9)	1236 (28.3)	<.001
IVD	247 (16.5)	300 (21.6)	124 (14.4)	<.001	671 (17.9)	1205 (27.6)	<.001

Abbreviations: CHF, congestive heart failure; COPD, chronic obstructive pulmonary disease; IQR, interquartile range; ISP, increased social pressure; IVD, ischemic vascular disease; LA, loss aversion; LBS, larger bonus size; NA, not applicable; RCT, randomized clinical trial.

^a^ Percentages have been rounded and may not total 100.

^b^ Includes 1 cardiologist.

^c^ Includes 1 pulmonologist.

^d^ Includes 1 cardiologist and 1 pulmonologist.

**Table 2.** Unadjusted Evidence-Based Quality Measure Achievement

Study Measure^a^	Randomized Controlled Trial						Cohort Study			
	2016-2015 Difference			Adjusted Pairwise *P* Value, 2016 vs 2015^b^			2016-2015 Difference		Differences-in-Differences	Adjusted Pairwise *P* Value^b^
	LBS Plus ISP	LBS Plus LA	LBS Only	LBS Plus ISP vs LBS Plus LA	LBS Plus ISP vs LBS Only	LBS Plus LA vs LBS Only	LBS	Non-LBS		
Overall, %	4.4	3.8	4.2				4.1	2.0	2.1	
Asthma, %										
Action plan	5.2	8.9	−0.9	>.99	>.99	>.99	4.3	4.1	0.2	>.99
Control treatment assessed	7.8	7.6	−0.1	>.99	>.99	>.99	5.4	7.3	−1.9	.95
Medication management	2.2	−0.5	0.0	>.99	>.99	>.99	1.1	6.7	−5.6	>.99
Adult BMI, %	−0.5	−1.6	3.5	>.99	>.99	.73	−0.1	−2.3	2.2	.12
Blood pressure										
Control (<140/90 mm Hg), %	0.8	0.4	5.2	>.99	>.99	>.99	1.6	−4.3	5.9	<.001
IVD/CAD measurement, %	−0.4	−0.3	3.2	>.99	.32	.03^b^	0.3	−1.0	1.4	.16
COPD spirometry evaluation, %	11.5	8.7	6.0	>.99	>.99	>.99	8.2	2.8	5.4	.08
Diabetes, %										
Eye examination	5.1	8.7	7.0	>.99	>.99	>.99	6.8	1.6	5.2	.16
Foot examination	15.5	−1.2	3.2	.91	>.99	>.99	7.5	0.4	7.1	<.001
HbA_1c_, %										
Control (<8%)	3.5	5.3	12.5	>.99	>.99	>.99	5.8	−0.5	6.3	.08
Poor control (>9%)	4.7	3.2	10.3	>.99	>.99	>.99	5.2	−0.5	5.7	.09
Testing	−2.1	0.1	3.8	>.99	>.99	>.99	−0.3	−1.3	1.0	>.99
Diabetes: medical attention for nephropathy, %	0.0	−0.6	0.0	>.99	>.99	>.99	−0.2	−0.4	0.2	>.99
CHF appropriate outpatient medication, %										
ACEI or ARB	12.1	1.2	−0.2	>.99	>.99	>.99	5.0	5.0	0.5	>.99
β-Blocker	46.2	3.8	14.7	.15	>.99	>.99	22.3	2.1	20.2	.70
IVD/CAD use of antiplatelet medication, %	10.8	1.6	1.1	>.99	>.99	.98	4.8	1.5	3.3	>.99
Depression screening and follow-up plan, %	6.2	1.6	3.5	.80	>.99	>.99	3.9	4.9	−1.1	>.99
Documentation of designated decision maker for medical care form, %	35.6	32.0	24.5	>.99	>.99	>.99	32.6	27.9	4.7	.17
Tobacco use, %										
Cessation counseling	4.8	9.2	2.9	>.99	>.99	>.99	6.5	−1.1	7.6	.04
Assessment	1.4	1.0	0.2	>.99	>.99	>.99	1.0	0.0	1.0	>.99

Abbreviations: ACEI, angiotensin-converting enzyme inhibitor; ARB, angiotensin II receptor blocker; BMI, body mass index; CAD, coronary artery disease; CHF, congestive heart failure; COPD, chronic obstructive pulmonary disease; HbA_1c_, hemoglobin A_1c_; ISP, increased social pressure; IVD, ischemic vascular disease; LA, loss aversion; LBS, larger bonus size.

^a^ Results indicated by % are reported as percentage point differences.

^b^ Reported *P* values for pairwise comparisons of the primary outcome of change in proportion of applicable chronic disease and preventive evidence-based measures meeting or exceeding benchmarks at the patient level use the Holm-Bonferroni correction. Multiple imputation was used for the approximately 11% of participants missing follow-up quality metric scores.

### RCT Testing Addition of ISP and LA to LBS

**Figure 2.**
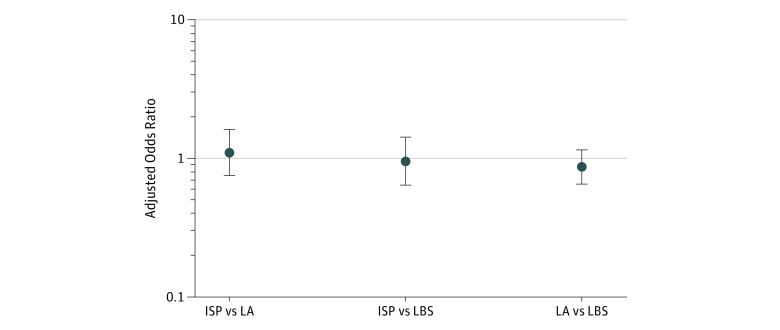
Adjusted Analysis of Evidence-Based Quality Measure Achievement in the Randomized Clinical Trial Comparison groups include larger bonus size (LBS) plus increasing social pressure (ISP), LBS plus loss aversion (LA), and LBS only for 2015 through 2016. Data are expressed as adjusted odds ratios with 95% CIs (error bars) for pairwise comparisons. The adjusted model includes the covariates consisting of patient demographics (age, sex, race, and the number of chronic disease registries in which a patient is included) and physician demographics (age, sex, tenure, and specialty). Pairwise difference-in-differences comparisons indicate no significant difference.

Patients in all groups experienced an increase in the mean rate of receiving evidence-based care. Patients in the LBS-only group had an absolute increase of 4.2 percentage points (87.6% in 2015 to 91.8% in 2016) (Table 2 and eTable 2 in Supplement 2). Patients in the LBS plus LA group had an increase in the mean rate of 3.8 percentage points (83.9% in 2015 to 87.7% in 2016) and patients in the LBS plus ISP group had an increase of 4.4 percentage points (84.6% in 2015 to 89.0% in 2016). However, adjusted pairwise testing revealed no differences between groups, with intervention group point estimates less than those of the control group (LBS plus LA group vs the LBS-only: aOR, 0.86 [95% CI, 0.65-1.15; *P* = .31]; LBS plus ISP vs LBS-only: aOR, 0.95 [95% CI, 0.64-1.42; *P* = .81]; and LBS plus ISP vs LBS plus LA aOR, 1.10 [95% CI, 0.75-1.61; *P* = .62]) (Figure 2). Analysis of individual measures did not reveal any systematic pattern of changes between the groups (Table 2). Sensitivity analyses gave similar results (eFigures 5-8 in Supplement 2).

### Cohort Study Testing LBS

**Figure 3.**
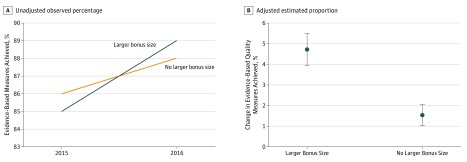
Analysis of Evidence-Based Quality Measure Achievement in Cohort Study The cohort study evaluated larger bonus size (LBS) vs non-LBS groups from 2015 through 2016. A, Observed (unadjusted) changes in the primary outcome for the LBS group compared with the non-LBS group. B, Estimated risk-adjusted changes in the primary outcome for the LBS group compared with the non-LBS group. Error bars indicate 95% CIs.

Patients in the LBS cohort experienced an increase in the mean rate of receiving evidence-based care of 4.1 percentage points (85.0% in 2015 to 89.2% in 2016) compared with an increase of 2.0 percentage points (86.2% to 88.2%) (Table 2 and eTable 3 in Supplement 2) in patients in the matched non-LBS group (Figure 3). Adjusted analysis demonstrated a significant association between the LBS cohort and increased evidence-based care (aOR, 1.25; 95% CI, 1.16-1.35; *P* < .001) or an estimated adjusted absolute increase of 3.2 percentage points (95% CI, 1.9-4.5 percentage points; *P* < .001). Sensitivity analyses provided similar results (eFigures 8 and 9 in Supplement 2).

Analysis of individual measures demonstrated that the associated increases in evidence-based care were significant for the following 3 measures (all other measures without significant changes) in the LBS vs non-LBS groups: blood pressure control (1.6–percentage point increase vs 4.3–percentage point decrease; *P* < .001), receiving a foot examination with a diabetes diagnosis (7.5– vs 0.4–percentage point increase; *P* < .001), and cessation of tobacco use (6.5–percentage point increase vs 1.1–percentage point decrease; *P* = .04) (Table 2).

### Physician Survey Results

Twenty-seven physicians (81.8%) responded to the preintervention survey and 32 (97.0%) to the postintervention survey. Although physicians in the LBS plus LA group indicated an increase in financial salience of the incentives (increase of 0.67 point on Likert scale [*P* = .04] vs −0.25 point for LBS-only group [*P* = .33] and 0.01 point for LBS plus ISP group [*P* = .41]), concerns about negative unintended effects also increased (change of 0.48 point [*P* = .01] vs 0.27 for LBS-only group [*P* = .14] and 0.11 point for the LBS plus ISP group [*P* = .25]) (eTable 3 in Supplement 2). The LBS plus ISP group indicated a decrease in teamwork (change of −0.37 point [*P* = .02] vs 0.03 point for LBS-only group [*P* = .48] and 0.18 point for LBS plus LA group [*P* = .30]). There were no detected harms to physicians and patients.

## Discussion

In this study testing behavioral economic principles in the P4P design through an RCT and evaluating increased bonus sizes, we found an increase in bonus size was associated with significantly improved quality for patients receiving care for chronic disease relative to a comparison group during a single year. Adding LA and ISP did not lead to further quality improvements, although attrition and a small sample size limited statistical power. We made 3 important findings.

First, an increase in the maximum bonus size of approximately $3355 (a mean of $16 per patient) or 32% per physician was associated with a small but significant improvement in evidence-based care received by patients. This improvement is particularly notable because the Advocate P4P program already had relatively large bonuses to start, with a mean of approximately $10 000 per physician per year for panel sizes of approximately 200 patients. A critique of P4P programs has been inadequate bonus sizes, with prior studies limited to specific settings that are less representative of general practice.^6,7,8^ Our results suggest that in a general primary care physician program with substantial bonus sizes and a large budget, further increases in bonuses were associated with gains in quality. However, they should also be interpreted with caution given a unique, single-institution setting in which the group exposed to LBS was lower performing and may have had a greater opportunity to improve. Further, because comparison group physicians may not have been as aware of their inclusion in the study, results may have been confounded by the Hawthorne effect.

Second, the addition of behavioral economic principles of LA and ISP did not increase the incentives’ effectiveness. However, the final sample size of 33 physicians was small, and thus the study was underpowered to detect clinically meaningful effects (noticeable in large 95% CI ranges), although the point estimates did not indicate directional improvements for either intervention group relative to the control group. Further, it is important to understand why the LA arm was not effective, in that only 2 physicians withdrew money from their virtual accounts. This outcome was despite low risk of having to pay back overdrawn dollars, because 31 physicians (93.9%) earned larger bonuses than the amount placed into virtual accounts in the previous year. These design features are important given that national policies are now using LA. The virtual bank accounts used herein were quite different than placing reimbursement at risk (such as the Merit Incentive Payment System) or prepaying dollars in advance (such as Medicare’s Comprehensive Primary Care Plus for 2965 practices nationwide).^25^ Although stronger forms of LA have been successful,^26^ the virtual account approach used herein was softer and, as it turns out, less effective.

Third, the results on group performance–based incentives are also important, given increasing interest in their use.^25,26^ The group incentives in this study were shared across a group of physicians in the same organization who did not necessarily share resources or work on the same care team, unlike in a prior study.^9^ The intention was to test ISP alone, rather than ISP mixed with incentives for teamwork directly. One other RCT that evaluated smaller incentives at the group level^9^ tested practice-level incentives (including nonphysicians) for improving adherence to hypertension guidelines within the Veterans Affairs system and found no benefit to group vs individual incentives.

### Limitations

This study has several limitations. First, this trial was conducted at a single health system network with a small sample size, dropout, and potential for confounding from the Hawthorne effect. However, these limitations represent the pragmatic nature of the study, because many physician networks and health plans face these challenges, and we used intention to treat and multiple imputation methods to mitigate bias. Second, the interventions may not have been strong enough or active long enough to drive behavior changes. Third, the evaluation of increased bonus size was an observational analysis subject to confounding.

## Conclusions

In a P4P program for physicians caring for patients with chronic disease, increasing bonus sizes was associated with improvements in quality, whereas adding an opportunity for LA and the proportion of incentive based on group performance did not lead to additional benefit. Further refinement of applications of behavioral economic principles in P4P design should be tested with larger sample sizes.

## Notes

Supplement 1. **Trial Protocol**

Supplement 2. **eFigure 1.** Loss Aversion With Larger Bonus Size Arm **eFigure 2.** Increased Social Pressure With Larger Bonus Size Arm **eFigure 3.** Common Support Graph for First Level of Matching With Trend Data **eFigure 4.** Common Support Graph for Second Level of Matching **eFigure 5.** Sensitivity Analysis for RCT Without Physician Fixed Effect Clustering at Group Practice Level **eFigure 6.** Sensitivity Analysis for RCT Without Imputation (Using Complete Case Data) **eFigure 7.** Sensitivity Analysis for RCT With Physician Random Effect **eFigure 8.** Sensitivity Analysis of Cohort Study Without Imputation (Using Complete Case Data) **eFigure 9.** Sensitivity Analysis of Cohort Study Without Physician Fixed Effects **eTable 1.** Measures in Composite Quality Measure Score for Chronic Disease Patients **eTable 2.** Complete Unadjusted Results of Randomized Clinical Trial **eTable 3.** Complete Unadjusted Results of Cohort Study **eTable 4.** Sample Characteristics of Cohort Study for Larger Bonus Size Without Matching **eTable 5.** Test of Trends for Difference-in-Differences Model Results **eTable 6.** Results of Physician Survey Administered Before and After Intervention **eMethods 1.** Propensity Matching Methods and Graphs for the Area of Common Support **eMethods 2.** Test of Trend Methods

Supplement 3. **Data Sharing Statement**
