# Supplementary material for: Effect of Financial Bonus Size, Loss Aversion, and Increased Social Pressure on Physician Pay-for-Performance: A Randomized Clinical Trial and Cohort Study
Source: JAMA Netw Open. 2019 Feb 8;2(2):e187950. doi: 10.1001/jamanetworkopen.2018.7950 (PMC6484616; doi:10.1001/jamanetworkopen.2018.7950)
Supplement: Supplement 1. — Trial Protocol [file jamanetwopen-2-e187950-s001.pdf]

Supplemental 1. Study Protocol and Changes to Analysis Plan

Effect of Financial Bonus Sizes, Loss Aversion, and Increased Social Pressure on Physician Pay-for-Performance: A Randomized Trial and Cohort Study

Principal Investigators:

Amol S. Navathe, MD, PhD
Assistant Professor of Medicine and Health Policy, Perelman School of Medicine
Staff Physician, CHERP, Philadelphia VA Medical Center
Associate Director, Center for Health Incentives and Behavioral Economics
Senior Fellow, Leonard Davis Institute of Health Economics, The Wharton School
University of Pennsylvania

Ezekiel J. Emanuel, MD, PhD
Provost for Global Initiatives
Diane v S. Levy and Robert M. Levy University Professor
Chair of the Department of Medical Ethics and Health Policy
University of Pennsylvania

Kevin G. Volpp, MD, PhD
Founders President's Distinguished Professor
Director, Center for Health Incentives and Behavioral Economics
Division Chief, Health Policy, Department of Medical Ethics and Health Policy
Perelman School of Medicine
University of Pennsylvania

Supported by:

The Commonwealth Fund
Grant No. 20150753

And

The Robert Wood Johnson Foundation
Grant No. 73111

Table of Contents

STUDY TEAM ROSTER 4
PARTICIPATING STUDY SITES 6
PRÉCIS 7
STUDY PROTOCOL 9
1. Study Objectives 9
1.1 Primary Objective 9
1.2 Secondary Objectives 9
2. Background on Behavioral Economics, Physician Incentives, and Primary Study Focus 9
2.1 Background 9
2.2 Study Rationale 10
3. Study Design 11
4. Selection and Enrollment of Participants 13
4.1 Inclusion Criteria 13
4.2 Exclusion Criteria 13
4.3 Study Enrollment Procedures 13
5. Study Interventions 13
5.2 Handling of Study Intervention 14
6. Study Procedures 14
6.1 Study Timeline 15
6.3 Description of Evaluation 15
7. Safety Assessments 15
7.1 Safety Monitoring 15
8. Statistical Considerations 15
8.1 General Design and Sample Size 16
8.2 Sample Size and Randomization 16
8.3 Outcomes 16
8.4 Data Analyses 17
9. Data Storage, Privacy, and Disclosure 17
9.1 Data Storage 17
9.2 Privacy 18
9.3 Data Disclosure 18

10. Participant Rights and Confidentiality 18
10.1 Institutional Review Board (IRB) Review 18
10.2 Informed Consent Forms 18
10.3 Study Discontinuation 18
11. Publication of Research Findings 19
SUMMARY OF CHANGES TO ORIGINAL PROTOCOL 20
Changes before the start of the intervention period: 20
Changes after the start of the intervention period: 21
PHYSICIAN SURVEY - PRE 22
PHYSICIAN SURVEY - POST 24
ADDITIONAL METHODS FROM PAPER 26
Pre-Trial Exclusions 26
Data Abstraction 26
Bootstrapping for Risk-Standardized Primary Outcome Measures 26
DEFINITION OF CLINICAL INTEGRATION SCORE 27
REFERENCES 28

STUDY TEAM ROSTER

University of Pennsylvania

Amol S. Navathe, MD, PhD. (Co-Principal Investigator), Department of Medical Ethics and Health Policy, University of Pennsylvania, 423 Guardian Drive, 1108 Blockley Hall, Philadelphia, PA 19104, Office: 215-573-4947, amol@wharton.upenn.edu

Ezekiel J. Emanuel, MD, PhD (Co-Principal Investigator), Department of Medical Ethics and Health Policy, University of Pennsylvania, 423 Guardian Drive, 14 Blockley Hall, Philadelphia, PA 19104, Office: 215-898-7226, zemanuel@upenn.edu

Kevin Volpp, MD, PhD (Co-Principal Investigator), Department of Medical Ethics and Health Policy, University of Pennsylvania, 423 Guardian Drive, 1120 Blockley Hall, Philadelphia, PA 19104, Office: 215-573-0270, volpp70@wharton.upenn.edu

Judy Shea, PhD (Co-investigator), Department of Medicine, University of Pennsylvania, 423 Guardian Hall, 1229 Blockley Hall, Philadelphia, PA 19104, Office: 215-573-5111, sheaja@pennmedicine.upenn.edu

Dylan Small, PhD Department of Statistics, University of Pennsylvania, 464 Jon M. Huntsman Hall, 3730 Walnut Street, Philadelphia, PA 19104, Office: 215-573-5241, dsmall@wharton.upenn.edu

Amelia Bond, PhD, Department of Health Care Management, The Wharton School, University of Pennsylvania, 3641 Locust Walk, Philadelphia, PA 19104, ambond@wharton.upenn.edu

Kristen Caldarella, MHA, Department of Medical Ethics and Health Policy, University of Pennsylvania, 423 Guardian Drive, 1138 Blockley Hall, Philadelphia, PA 19104, Office: 215-573-4934, kcald@pennmedicine.upenn.edu

Shireen Matloubieh, MPH, Department of Medical Ethics and Health Policy, University of Pennsylvania, 423 Guardian Drive, 1136 Blockley Hall, Philadelphia, PA 19104, Office: 215-573-6742, smatlo@pennmedicine.upenn.edu

Zoe Lyon, BA, Department of Medical Ethics and Health Policy, University of Pennsylvania, 423 Guardian Drive, 1136 Blockley Hall, Philadelphia, PA 19104, Office: 215-573-2688, zlyon@pennmedicine.upenn.edu

Jingsan Zhu, MBA, MS, Department of Medical Ethics and Health Policy, University of Pennsylvania, 423 Guardian Drive, 1113 Blockley Hall, Philadelphia, PA 19104, Office: 215-573-9731, jingsan@pennmedicine.upenn.edu

Akriti Mishra MS, Department of Medical Ethics and Health Policy, 423 Guardian Drive, 1121 Blockley Hall, Philadelphia PA, 19104, Office: 215-573-5654, akritim@pennmedicine.upenn.edu

New York University

Andrea Troxel, ScB, (Co-Investigator), Department of Population Health, New York University, 650 First Avenue, Fifth Floor 521, New York, NY 10016, Office: 212-263-6527, andrea.troxel@nyumc.org

Advocate Health Care

Lee Sacks, MD, Advocate Aurora Health, 3075 Highland Parkway, Suite 600, Downers Grove, IL 60515, Office: 630-929-8707, lee.sacks@advocatehealth.com

Pankaj Patel, MD, Advocate Physician Partners, 3075 Highland Parkway, Suite 600, Downers Grove, IL 60515, Pankaj.patel@advocatehealth.com

Carrie Nelson, MD, MS, FAAFP, Advocate Physician Partners, 1701 Gold Road, Suite 2-1100, Rolling Meadows, IL 60008, Office: 847-635-4196, carrie.nelson@advocatehealth.com

Don Calcagno, MBA, Advocate Physician Partners, 1701 Golf Road, Rolling Meadows, IL 60008, Office: 847-635-3396, don.calcagno@advocatehealth.com

Salvatore Vittore, Advocate Physician Partners, 1701 Golf Road, Suite 2-1100, Rolling Meadows, IL 60008, Office: 847-635-3378, Salvatore.vittore@advocatehealth.com

Kara Sokol, Advocate Physician Partners, 1701 Golf Road, Rolling Meadows, IL 60008, Office: 847-699-4306, kara.sokol@advocatehealth.com

Kevin Weng, Advocate Physician Partners, 1701 W. Golf Road, Suite 2-1100, Rolling Meadows, IL 60008, Office: 847-699-4305, kevin.weng@advocatehealth.com

Nichia McDowald, Advocate Physician Partners, 1701 Golf Road, Suite 2-1100, Rolling Meadows, IL, 60008, Nichia.mcdowald@advocatehealth.com

Paul Crawford, MD, Advocate Physician Partners, 10725 South Western, Chicago, Illinois 60643, Office: 708-952-3040, paul.crawford@advocatehealth.com

PARTICIPATING STUDY SITES

Advocate Physician Partners: Pankaj Patel, MD, Advocate Physician Partners, 3075 Highland Parkway, Suite 600, Downers Grove, IL 60515, Pankaj.patel@advocatehealth.com

PRÉCIS

Study Title
A Pragmatic Policy Trial Testing Larger Bonus Sizes and the Behavioral Economic Principles of Loss Aversion and Increased Social Pressure in Physician Pay-for-Performance

Objectives
The key goal of this randomized trial is to test whether using behavioral economic principles in addition to larger bonus sizes in the structure of provider incentives and the practice environment may improve provider performance, especially in settings that are moving away from fee-for-service reimbursement and fragmented care towards new payment and delivery models that emphasize coordination of care and provider accountability. We also directly evaluate the effect of increasing bonus sizes in an accompanying non-randomized study.

Design and Outcomes
This research project will conduct a prospectively designed experimental evaluation of the impact of social pressure by varying individual and group incentives and the comparative effectiveness of endowment loss aversion on provider physician performance via a multi-arm experiment and a prospectively designed, non-randomized observational study of the impact of increasing bonus sizes. We will complement the quantitative RCT evaluations with pre- and post-intervention qualitative surveys of physicians and patients to better understand the influence on behavior change, culture, acceptability of the incentive program, and patient reported outcomes on health and experience. These evaluations will contribute toward an empirical foundation for informing the re-design of existing physician incentive programs and implementation of new policies, by evaluating the impact of promising behavioral economics principles in improving quality metrics and patient experience in the context of provider payment – above and beyond increasing bonus sizes themselves. The examination of the effect of larger bonus sizes will be conducted in a separate but related observational analysis, since all physicians in the randomized trial will receive larger bonus sizes, using a design that evaluates changes in the 3 intervention arms combined compared to a group of propensity matched physicians who did not receive an increase in bonus size.

Interventions and Duration
Experimentally evaluate the impact of *social pressure* and the effectiveness of endowment *loss aversion* on physician performance in addition to a larger bonus size.

Utilize an observational, quasi-experimental design to assess the impact of providing a larger bonus size in the P4P program.

This multi-arm Randomized Controlled Trial (RCT) will compare the effectiveness of endowment loss aversion incentive design and increased group incentives to enhance social pressure for physician financial incentives in improving quality of care. Quality of care will be

measured using the specified HEDIS-based patient-level metrics. The intervention will consist of an active phase of 12 months and sites will be randomized equally to two interventions and an "active control" arm (Arm 1) with the existing incentive design but larger bonus size. The intervention arms 2 (endowment loss aversion) and 3 (social pressure) will build off of the current incentive program. Arm 2 will change the framing of incentives to physicians from the existing framing to a potentially more powerful loss aversion method called 'the endowment effect.' Arm 3 will increase the percentage of the individual physician incentive based on his or her group practice's performance to 50% from the 30% in the current program (this will correspondingly decrease the 70% paid based on individual performance to 50%). All arms will include cluster randomization of physicians by site.

We will select and randomize Advocate practice sites from the Trinity PHO equally to the three study arms (Arms 1 – 3) described above. Baseline data will be drawn for the years 2014-2015 for the physicians in each aim with the active phase of the trial starting on January 1, 2016 and running for 12 months.

All participants will be tracked at baseline at 3, 6, 9, and 12 months during the interventions and provided periodic Proformas (Figure 1 and 2) with performance on specific metrics and overall.

This will be accompanied by an observational analysis compared all patients and physicians included in the RCT with patients of propensity-matched physicians who did not receive a larger bonus size or participate in the randomized trial. Physicians will be matched on demographics as well as pre-trial performance level and pre-trial trend in performance.

Sample Size and Population

We anticipate enrolling approximately 20 practice sites and 40 physicians and 1400 patients per group. Randomization will occur at the level of the physician practice. We assume an average office size of 2, and a conservative intraclass correlation (ICC) estimate of 0.25. We wish to be able to detect a clinically meaningful increase in quality metric score achievement of at least 5 percent between the control arm and either the endowment loss aversion group or the increased social pressure group. Using 80% power to detect differences in the change in proportion of evidence based measures received between any incentive group and control of 5% will require approximately 3,420 participants (1,140 per group).

The observational analysis will utilize a matched design, therefore we anticipate analyzing an additional 20 practice sites and 40 physicians in comparison to those enrolled in the RCT.

STUDY PROTOCOL

1. Study Objectives

1.1 Primary Objective

Experimentally evaluate the impact of social pressure and the effectiveness of endowment loss aversion on physician performance above and beyond larger bonus size.

Use a complementary, prospective observational, quasi-experimental design to directly evaluate the impact of larger bonus size.

1.2 Secondary Objectives

Evaluate how practice behavior changes by looking at pre and post survey data.

Evaluate patient satisfaction to assess their satisfaction with their physician throughout the trial.

2. Background on Behavioral Economics, Physician Incentives, and Primary Study Focus

2.1 Background

The American health care system is undergoing tremendous transition. The objective is to control total health care costs while improving or maintaining quality of care. A fundamental aspect of the transition is payment reform. While fee-for-service payment (FFS) remains the dominant form of payment, the last few years have seen a major shift toward alternative payment methods as highlighted by Secretary Burwell's recent announcement that Medicare and Medicaid will be rapidly moving away from FFS in the next few years.[1] There are now hundreds of organizations, such as hospitals and large physician groups functioning as accountable care organizations (ACOs) and participating in bundles (e.g. the CMS Bundled Payment for Care Improvement (BPCI) program), that are participating in shared-savings payment programs and "two-sided risk contracts," such as in the CMS Pioneer ACO program. At the opposite end of the payment spectrum from FFS are fully capitated delivery systems, of which there are relatively few. While they differ in exact mechanisms, these models share a common strategy: tying provider (physician, hospital, health system) reimbursement to performance on costs and outcomes. Furthermore, they have been paired with powerful regulatory concessions by relaxing aspects of Stark Laws and Anti-kickback statutes to allow for gainsharing with physicians.

Optimal provider payments will vary not only with an organization's position along the payment spectrum but also as a function of its particular mission, culture, local competitive environment,

patient population, and contractual and financial relationship with providers. To promote appropriate high-quality utilization informed by evidence-based guidelines, payers in FFS environments have often supplemented their payment mechanisms with pay-for-performance (P4P) strategies. But to date, P4P has demonstrated little effect on physician behavior.[2-5] This result probably reflects the relatively small size of the financial incentives employed in most pay-for-performance programs to date, but may also reflect important design limitations.

Traditional P4P and most other provider payment programs have been developed through trial-and-error, partially informed by the best science of human motivation, such as behavioral economics. Behavioral economics has revealed systematic ways in which human behavior is shaped not merely by the size of incentives, but also by their design and how they are delivered.

2.2 Study Rationale

There have been limited efforts to experimentally test ways to improve on P4P programs with little, if any, consideration of behavioral economics. These have included mostly retrospective analysis of demonstration projects and only few randomized controlled trial (RCT) of physicians in a similar policy context to date.[6-9] In the most relevant trial, individual incentives were compared with team-based, practice level incentives and a combined incentive program; individual incentives were found to be most effective in adherence to hypertension guidelines. The maximum size of incentives for primary care physicians (PCPs) was approximately 1.6% of annual income, with nurse team members receiving above $500 in incentives in the team and combined arms.16 Effects were modest and did not persist beyond 12 months. This study did not incorporate the behavioral economic principles described above in altering how incentives were framed or paid, how performance was communicated, utilizing social comparisons between providers, or the use of goal gradients.

In general, efforts to reform provider payment have been built on the assumption that providers are largely rational and have not utilized insights from behavioral economics.[10, 11] The principles of behavioral economics have been successfully used in the design of patient incentives for smoking, substance abuse, obesity, and drug adherence.[12-20] Less is known about designing incentives to influence physician care patterns. This could also partly explain the lackluster results of many existing efforts aimed at designing physician incentives to promote high-quality, high-value care.

Behavioral economics can provide insights into how to improve the effectiveness of physician incentives to deliver higher quality and lower cost care. Its principles can be implemented through creative design of incentives that can be tested in the context of provider incentives with insights into decision errors that can be leveraged to improve the effectiveness of financial incentives. Examples include unbundling incentives from other payments to make them more salient (mental accounting), designing incentives to provide immediate vs. more delayed rewards due to the importance of immediate gratification, loss framing, and avoidance of choice

overload. Behavioral economics has also emphasized thoughtfully structuring the choice environment and the use of non-financial rewards and penalties to shape behavior. By applying these behavioral economics principles to physician incentives, policymakers, payers, and health systems alike could improve the effectiveness of incentives by making them more salient to physicians and better aligned with performance goals, without increasing the overall allocation of funds for incentive payments. For example, the ACO and BPCI gainsharing policy where incentives are provided at the organization level, could be an important target for incentive re-design. However, to what extent applying behavioral economic principles versus increasing bonus sizes to be more significant, and whether these have synergistic effects, is unknown.

3. Study Design

Based on recommendations from the academic team members and health system partner, we will pursue a staged approach to further develop our initiative using behavioral economics and provider payment. Arm 1 will serve as the 'active control' in which physician incentives payments are 70% individual / 30% group-based and delivered with the current design, though with a larger bonus size (increase of ~32% on average). Arm 2 will test loss aversion by creating an endowment effect for the physician incentives, keeping the 70% individual / 30% group-based components constant. This will be done by giving providers 50% of the bonus at the beginning of the year in a virtual account and making retention of incentive dollars conditional on performance. This would provide an alternative to the once-yearly payment currently provided by Advocate. The incentive amount (bonus size) will also be increased similarly by ~32% in this Arm. Arm 3 will test enhanced social pressure by increasing the group-based performance component of the individual physician's incentive payment to 50% (with other 50% based on individual performance) and will keep the other incentive design constant, along with the larger bonus size. In all Arms 1-3, the physicians will receive an additional 32% or approximately $3500 more incentive dollars available (this will be provided across all 155 physicians in the Trinity Physician-Hospital Organization regardless of participation in the study).

The experiment will be conducted in the Trinity Physician-Hospital Organization (PHO), a member of Advocate Physician Partners because Advocate Physician Partners has already implemented a performance incentive program and are very interested in improving their incentive program. Furthermore, Trinity is a lower performing PHO within Advocate, without obvious explanatory factors for the discrepant results, for which the leadership is very interested in trying new methods of incentive design to improve quality of care. It comprises 164 Trinity physicians eligible for incentive distribution for the 2014 performance year, of which 155 are affiliated physicians and 9 are employed physicians. These physician provided care through over 35,000 unique outpatient encounters in 2014. The Trinity PHO ranks last amongst the 10 Advocate PHOs in physician –level quality score attainment (as measured by Advocate), with achievement of 69% of the possible score while the next highest PHO attains 74% and the highest achieves 91%; the mean score is 86.7%. Within the Trinity PHO, there is significant

variation in physician performance with mean score (as a percent) of 69% with a standard deviation of 12.8%, and a range from 37.6% to 100%. Furthermore, the patient satisfaction with outpatient visits trails that of other PHOs, with Trinity physicians in the 23rd percentile nationally versus 48th percentile across the other PHOs. The physicians are distributed across 81 practices with 65 solo practices and 10 practices of 4 physicians or more.

Quality of care will be measured using the metrics described in Table 1. The intervention will consist of an active phase of 12 months and physicians will be randomized equally to two interventions and an "active control" arm (Arm 1). Specifically, randomization will occur at the physician practice site level and the randomization will be stratified by specialty vs. primary care, prior performance (low vs. not low) and practice site size (solo vs. multi-physician). Baseline data will be drawn for the years 2014-2015 for the physicians in each aim with the active phase of the trial starting on January 1, 2016 and running for 12 months.

The data for the physician scores will be readily available given that the performance incentive program being studied already exists and the experiment will only modify the way physicians receive financial incentives or modify how those incentives are calculated. We will capture the data through Advocate's platform for administering the program, which includes reporting tools and software to incorporate Cerner electronic medical records (EMR) and population health management (PHM) data along with pharmacy data.

Patient reported data will be collected directly from Advocate leveraging the existing survey infrastructure in place for quality measurement. Advocate currently receives raw survey results at the patient-question level from the survey administrator Press Ganey, Inc. Press Ganey mails a paper survey to each patient after every visit to an Advocate affiliated or employed physician and codes the results in an electronic database. The survey tool utilized will be the Clinician and Group Consumer Assessment of Healthcare Providers and Systems (CG-CAHPS) survey. To increase the response rate higher than 22 percent, we will provide a $15 participation incentive to each patient who returns a completed survey. A random sample of 1500 unique patient visits (from over 30,000) will be recruited, stratified across practice sites, physicians, and specialties. This patient survey will be administered by the Advocate HealthCare vendor Professional Research Consultants.

Physician surveys will be administered via Survey Monkey by Advocate, which is Advocate's usual process for surveying physicians.

Penn's Health Services Research Data Center (HSRDC) will serve as the coordinating center for data acquisition and protection for all data. All data will be transmitted to the HSRDC by secure FTP.

4. Selection and Enrollment of Participants

4.1 Inclusion Criteria

We will include all Advocate practice site that have the following characteristics: participation in the Trinity PHO that participates in the Advocate Clinically Integrated Network (CIN), one or more full-time physician, participation in the incentive program, use of Cerner EHR and registry function, and participation in Press Ganey survey program. Practices will be included regardless of average patient panel size, average patient complexity and heterogeneity, geographic/zip code demographic and socioeconomic characteristics, and primary care only vs. multi-specialty group. Physicians who were affiliated with Advocate, but not employed by Advocate, will be included. Physicians with uniquely attributed patients with one of five chronic diseases (asthma, chronic obstructive pulmonary disease, diabetes, coronary artery disease or ischemic vascular disease, congestive heart failure) will be able to participate, with preference given to primary care physicians if there is shared attribution with specialists (i.e., patients will be uniquely attributed to physicians). Only patients with one of the five chronic diseases will be included.

4.2 Exclusion Criteria

Sites will be excluded if they do not use an EHR, have not participated in quality reporting, have not practiced with Advocate for the entire pre-intervention period, have never been part of a pay-for-performance incentive program in the past, or do not have eligible patients. Patients will be excluded if they have not been attributed to an Advocate Physician Partners physician for more than twelve continuous months.

4.3 Study Enrollment Procedures

Patients will first be assigned to a practice site based on the location of their elected PCP, or in the event of no election to the practice site with the greatest number of EMR encounters for that patient. Furthermore, patients will be attributed to their elected PCP or the physician at the assigned site with the greatest number of EMR encounters for that patient. This will allow for inclusion of specialists when a patient does not have an elected PCP and the greatest number of EMR encounters is to a specialist physician. Because sites will be randomized (not patients or providers), there will be clear delineation of a patient to a provider in each arm with no confusion when a patient sees multiple providers. We are requesting a waiver of written informed consent for physicians since there is no appreciable risk to physicians in participating (the purpose of the incentive program changes is to increase quality and incomes).

5. Study Interventions

5.1 Interventions, Administration, and Duration

The intervention period will be 12 months in length for all participants.

5.2 Handling of Study Intervention

The following interventions will be compared:

The *larger bonus size* intervention provided maximum P4P bonuses larger than previous years by $3,355 per physician, representing an approximate 32 percent increase in bonus size for the average physician. Quality metrics and scoring methodology will be left unchanged. This intervention represents an 'active control' in which the physicians will receive larger bonuses than physicians not participating in the RCT (and larger than they themselves received the prior year); no additional feedback on performance on financial expectations will be provided other than the year-end proforma as per standard Advocate practice.

The *loss aversion plus larger bonus size intervention* will include the larger maximum bonus plus pre-funded incentives in a virtual health system bank account in the physician's name. The pre-funded incentives, which are 50% of the expected incentives based on prior year performance, will be placed into the virtual account on January 1, 2016; physicians will be able to access these dollars by requesting them in writing by email or regular mail from the Advocate network chief financial officer (who usually sends out the bonus checks to them on a yearly basis). Physicians in this intervention group will receive four additional proformas (Figure 1) in February, July, September, and November of 2016 that indicate the total amount of pre-funded incentive dollars, the amount accessed year-to-date, the projected 2016 incentive bonus size based on current performance, and the residual unearned incentive.

The *increased social pressure plus larger bonus size* intervention will include the increased maximum bonus but also will change the composite quality score from 70% based on individual score and 30% based on PHO score (the average of all individual scores in the group as defined by PHO) to 50% individual and 50% group (here defined as all physicians in the same intervention group). Physicians in this intervention group will also receive four additional proformas (Figure 2) on the same dates as above that indicate the additional P4P bonus dollars that would be earned by the 20 percentage point increase in the weighting given to group score as well as an unblinded list of physicians with performance scores on two of the quality measures. Physicians with scores below the performance threshold will be highlighted.

The comparison group for the observational, quasi-experimental comparison will not receive any changes to their incentive.

6. Study Procedures

6.1 Study Timeline

Study Task	Timeline
Period of Performance Begins	November 2015
Administer pre-trial qualitative surveys to physician participants	December 2015
Experiment begins	January 2016
Proformas sent to loss aversion and increased social pressure arms	January 2016
Proformas sent to loss aversion and increased social pressure arms	April 2016
Evaluate preliminary results	June 2016
Begin patient surveys	July 2016
Proformas sent to loss aversion and increased social pressure arms	August 2016
Proformas sent to loss aversion and increased social pressure arms	November 2016
Complete experiment	December 2016
Complete patient survey mailings	December 2016
Administer post-trial qualitative physician surveys	January 2017
Aim 2 Quantitative and Qualitative Analysis	February – April 2017

6.3 Description of Evaluation

We are not recruiting patients for this study, therefore we are requesting a waiver of consent. There is no harm to the persons whose data we are reviewing and it would be impossible to obtain consent on them at this point. The waiver of consent is being requested because the research presents no more than minimal risk to subjects and involves no procedures for which written consent is normally required outside of the research context.

7. Safety Assessments

7.1 Safety Monitoring

Safety monitoring per standard Advocate clinical practice and governance will occur under the standard quality improvement project protocols.

8. Statistical Considerations

8.1 General Design and Sample Size

We are proposing Advocate as our health system partner because it has implemented a performance incentive program that incorporates immediacy through an online registry that provides real-time feedback, who are organized into practice sites of differing size and proportion of Advocate patients (for affiliates). Furthermore, Advocate Physician Partners brings together more than 6,300 affiliated and employed physicians and 12 hospitals in the Advocate Health Care System, providing significant scale to the evaluation effort. We anticipate enrolling approximately 20 practice sites and 40 physicians and 1400 patients per group. Randomization will occur at the level of the physician practice. We assume an average office size of 2, and a conservative intraclass correlation (ICC) estimate of 0.25. We wish to be able to detect a clinically meaningful increase in quality metric score achievement of at least 5 percent between the control arm and either the loss aversion group or the increased social pressure group. Using 80% power to detect differences in the change in proportion of evidence based measures received between any incentive group and control of 5% will require approximately 3,420 participants (1,140 per group). The observational, quasi-experimental analysis will include an additional approximately 20 practice sites and 40 physicians.

8.2 Sample Size and Randomization

Eligible affiliated physicians in the RCT will be randomized by practice site to active control or two intervention groups in a 1:1:1 ratio, stratified by primary care versus specialist (family medicine, internal medicine, or pediatrics versus or other specialty that included cardiology, nephrology, or obstetrics and gynecology). Study participants and operational staff will not be blinded to group assignment, because knowledge of the incentives is essential to their mechanism, but study investigators and data analysts will remain blinded until all follow-up data are obtained and primary analyses are finalized.

8.3 Outcomes

8.3.1 Primary Outcome

The primary outcome will be the impact of physician performance on patient chronic disease quality metrics from baseline to 12 months. This will be a patient-level analysis of the proportion of applicable chronic disease and preventive evidence-based measures within the P4P program meeting or exceeding national HEDIS benchmarks at the patient level, representing a patient's view of the proportion of evidence-based care received. This primary outcome will apply to both the randomized trial and the observational study.

8.3.2 Secondary Outcome

Our secondary outcomes of interest include incentive payout and individual quality metrics within the composite.

8.4 Data Analyses

Our initial approach to analysis will be a descriptive comparison of site, physician participant, and patient attributes across the arms. Continuous variables will be described by means and standard deviations, or by medians and interquartile ranges if they appear non-normal (where appropriate, such variables will be transformed). The primary analysis will consist of an intent-to-treat approach using a linear regression analysis of the effect of treatment assignment on the outcome of change in the patient-level composite quality measure from baseline to 12 months. The patient-level composite quality measure score will reflect the same metrics to which financial incentives are tied for the chronic disease patients (Table 2). We will examine linear regression diagnostics using standard approaches to ensure appropriate model fit. Standard errors will be corrected for heteroscedasticity and clustered at the physician level. Additional exploratory analyses will use longitudinal models to assess the series of quality measures over time, to determine the shape of the trajectory and whether those trajectories differ by treatment group, though we do not anticipate any inter-group differences prior to the intervention. All hypothesis tests will be two-sided.

The primary analysis will consist of an unadjusted comparison of the change in patient-level composite quality metric score by treatment arm, using indicator variables for the three active treatments. An initial set of hypothesis tests will compare each active arm to the control arm, using a Bonferroni-corrected, two-sided p-value of 0.017 to determine statistical significance. We will conduct further testing of any treatment arms that show significant differences with the control. We will use generalized linear regression for all quality metrics. When performing analysis of Aims 2 and 3 we will also consider a subset of performance metrics within CI composite including chronic disease, population health/wellness, and screening categories as these are more likely to be influenced by practices within a 1 year timeframe. This will also enable us to evaluate heterogeneity in movement across measures.

The observational, matched quasi-experimental study will utilize a difference-in-differences design with the same primary outcome, control variables, and generalized linear model as the RCT analysis; however, because of a lack of randomization it will include physician fixed-effects and will utilize a set of matched APP physicians who did not participate in the RCT and are not part of the Trinity PHO. Propensity matching will be performed on physician demographics, 2015 performance, and the trend in performance for 2014-2015. Standard errors will be clustered at the patient level given multiple repeated measures at the patient level.[21]

9. Data Storage, Privacy, and Disclosure

9.1 Data Storage

All study data for this project will be stored on the secure/ firewalled servers of the HRSDC Data

Center, in data files that will be protected by multiple password layers. These data servers are maintained in a guarded facility behind several locked doors, with very limited physical access rights. They are also cyber-protected by extensive firewalls and multiple layers of communication encryption. Electronic access rights are carefully controlled by University of Pennsylvania system managers.

9.2 Privacy

We will receive de-identified data from Advocate Health. No patient PHI will be transferred, as all patient level data will be de-identified. Physicians will be tracked over time with a unique identifier. All of these data will be stored in an encrypted database that conforms to applicable data security standards.

9.3 Data Disclosure

The data will not be disclosed to anyone outside of the research team. De-identified data may be shared, if requested by The Commonwealth Fund, our sponsor, in the event of an audit, or the Office Human Research protections at the University of Pennsylvania.

10. Participant Rights and Confidentiality

10.1 Institutional Review Board (IRB) Review

The study protocol and the waiver of consent document will be reviewed and approved by the University of Pennsylvania's Institutional Review Board (IRB) and the Advocate Health System Institutional Review Board.

The Advocate Health System IRB initially approved the protocol and waiver of consent document on December 3, 2015.

The University of Pennsylvania's IRB initially approved the protocol and waiver of consent document on December 7, 2015.

10.2 Informed Consent Forms

There is a waiver of informed consent for physicians and patients.

10.3 Study Discontinuation

Advocate will follow its standard quality improvement procedures and discontinue per its protocols.

11. Publication of Research Findings

Publication of results from our research will follow the NIH Public Access Policy, which requires that we submit to the National Library of Medicine's PubMed Central an electronic version of final, peer-reviewed manuscripts upon acceptance for publication, to be made publicly available no later than 12 months after the official date of publication.

SUMMARY OF CHANGES TO ORIGINAL PROTOCOL

Changes before the start of the intervention period:

Original Protocol	Change to Protocol	Date of Change
Arm 3 named Social Pressure	Arm 3 named increased social pressure and larger bonus size	December 1, 2015
No changes to bonus size	All 3 arms to receive increased bonus size as a part of the observational study design	December 1, 2015
No observational study of larger bonus size	Prospectively design observational, quasi-experimental study added to directly test impact of larger bonus size	December 1, 2015
Primary outcome of physician level Advocate Clinical Integration composite score	The primary outcome will be the impact of physician performance on patient chronic disease quality metrics from baseline to 12 months. This will be a patient-level analysis of the proportion of applicable chronic disease and preventive evidence-based measures within the P4P program meeting or exceeding national HEDIS benchmarks at the patient level, representing a patient's view of the proportion of evidence-based care received. This primary outcome will apply to both the randomized trial and the observational study.	December 1, 2015
Include all patients	Include patients with one of five chronic diseases (asthma, chronic obstructive pulmonary disease, diabetes, coronary artery disease or ischemic vascular disease, congestive heart failure) – because data not available for all non-chronic disease patients in registry data	December 1, 2015
Randomize specialists	Randomize all specialists to receive intervention, but only include those with attributed patients who are not also attributed to a PCP in the analysis	December 1, 2015

Changes after the start of the intervention period:

Original Protocol	Change to Protocol	Date of Change
Arm 2 named endowment loss framing	Arm 2 re-named loss aversion plus larger bonus size	January 2016
Proformas to be sent to physicians in January	Proformas sent to physicians in February	January 2016
Proformas to be sent to physicians in April	Proformas sent to physician in July	April 2016
Patient surveys to be conducted by Press Ganey	Patients surveys to be conducted by Professional Research Consultants	April 2016
Proformas to be sent to physicians in August	Proformas sent to physicians in September	September 2016
Secondary outcome to analyze incentive payout	Did not analyze the incentive payout as a secondary outcome	January 2017

PHYSICIAN SURVEY - PRE

0. Baseline Qs on attitude toward financial incentives

1) Physicians should be rewarded when they provide higher quality care
2) Financial incentives for physicians are an effective way to improve the quality of health care
3) Financial incentives are more effective as an incentive compared to non-financial incentives such as peer-recognition
4) The 2015 CI program for physicians is an effective way to improve the quality of health care

I. Teamwork/Collaboration

5) I am able to get the cooperation of other physicians as needed to obtain the 2015 CI financial incentive
6) I am able to get the cooperation of support staff as needed to obtain the 2015 CI financial incentive
7) How effective are each of the following in improving the quality of care you provide to your patients?
    a. Teamwork or communication among physician or other medical care professionals is effective in improving the quality of care I provide.
    b. The level of patient access to preventative care and health education is effective in improving the quality of care I provide.
    c. Care coordination among other physicians and care managers for chronically ill patients is effective in improving the quality of care I provide

II. Financial Salience

8) The 2015 CI program represents an opportunity for me to increase my income
9) The 2015 CI program is sufficiently large to compensate for expenditures that might be necessary in order to meet the quality target
10) The timing of when I receive the 2015 APP CI incentive distribution makes me focus on improving my CI score
11) The portion of the 2015 CI financial incentive based on group/Trinity PHO performance increases my focus on improving my 2015 CI score.

III. Practice Environment/Support/Resources - including practice improvement, QI, IT, data

12) I am satisfied with my practice.
13) My practice makes more monetary and non-monetary resources available compared to last year.
14) Trinity PHO leadership invests extra time and effort to help me obtain the 2015 CI financial incentive.
15) My support staff invests extra time and effort to help me achieve the 2015 CI financial incentive.
16) My APP patients have adequate access to necessary ancillary services.
17) There are enough support staff in my practice.

IV. Awareness/Understanding + Acceptability/Control

18) I have adequate information about the scoring system used to compute the 2015 CI financial incentive amount
19) I get useful feedback regarding my progress toward improving my 2015 CI score.
20) Physicians within Trinity PHO are on a level playing field for obtaining the 2015 CI financial incentive.
21) The actions necessary to obtain the 2015 CI financial incentive are largely within my control.
22) Because of the clinical characteristics of my APP patients, it will be more difficult for me to obtain the 2015 CI financial incentive than it will be for other physicians within Trinity PHO.

V. Individual impact on clinical behavior

23) Because of the 2015 CI program, I invest extra time and effort in the care of my APP patients
24) Because of the 2015 CI program, I have changed my practice behavior to obtain this financial incentive.
25) Because of the 2015 CI program, overall, my APP patients are getting better care.
26) I would be just as focused on improving my CI score without 2015 CI financial incentive.
27) Obtaining the 2015 CI financial incentive brings me favorable recognition from my colleagues
28) Knowing my CI score helps me focus my time and effort constructively.

VI. Unintended Consequences

29) The effort required to obtain the 2015 CI financial incentive leads me to focus less on non-APP patients in my practice.
30) Efforts to obtain the 2015 CI financial incentive hinder me from providing other essential medical services to my APP patients.
31) The effort required to obtain the 2015 CI financial incentive has improved the care of non-APP patients in my practice

PHYSICIAN SURVEY - POST

<u>*0.* Baseline Qs on attitude toward financial incentives</u>

1) Physicians should be rewarded when they provide higher quality care
2) Financial incentives for physicians are an effective way to improve the quality of health care
3) Financial incentives are more effective as an incentive compared to non-financial incentives such as peer-recognition
4) The 2016 CI program for physicians is an effective way to improve the quality of health care

<u>I. Teamwork/Collaboration</u>

5) I was able to get the cooperation of other physicians as needed to obtain the 2016 CI financial incentive
6) I was able to get the cooperation of support staff as needed to obtain the 2016 CI financial incentive
7) How effective were each of the following in improving the quality of care you provide to your patients?
    a. Teamwork or communication among physician or other medical care professionals was effective in improving the quality of care I provide.
    b. The level of patient access to preventative care and health education was effective in improving the quality of care I provide.
    c. Care coordination among other physicians and care managers for chronically ill patients was effective in improving the quality of care I provide

<u>II. Financial Salience</u>

8) The 2016 CI program represented an opportunity for me to increase my income
9) The 2016 CI program was sufficiently large enough to compensate for expenditures that might be necessary in order to meet the quality target
10) The timing of when I received the 2016 APP CI incentive distribution made e me focus on improving my CI score
11) The portion of the 2016 CI financial incentive based on group/Trinity PHO performance increased my focus on improving my 2016 CI score

<u>III. Practice Environment/Support/Resources</u>

12) I am satisfied with my practice.
13) My practice made more monetary and non-monetary resources available compared to last year.
14) Trinity PHO leadership invested extra time and effort to help me obtain the 2016 CI financial incentive.
15) My support staff invested extra time and effort to help me achieve the 2016 CI financial incentive.
16) My APP patients had adequate access to necessary ancillary services.
17) There are enough support staff in my practice.

<u>IV. Awareness/Understanding + Acceptability/Control</u>

18) I had adequate information about the scoring system used to compute the 2016 CI financial incentive amount
19) I got useful feedback regarding my progress toward improving my 2016 CI score.
20) Physicians within Trinity PHO were on a level playing field for obtaining the 2016 CI financial incentive.
21) The actions necessary to obtain the 2016 CI financial incentive were largely within my control.
22) Because of the clinical characteristics of my APP patients, it was more difficult for me to obtain the 2016 CI financial incentive than it was for other physicians within Trinity PHO.

V. Individual impact on clinical behavior

23) Because of the 2016 CI program, I invested extra time and effort in the care of my APP patients
24) Because of the 2016 CI program, I changed my practice behavior to obtain this financial incentive.
25) Because of the 2016 CI program, overall, my APP patients received better care.
26) I would have been just as focused on improving my CI score without 2016 CI financial incentive.
27) Obtaining the 2016 CI financial incentive brought me favorable recognition from my colleagues
28) Knowing my CI score helped me focus my time and effort constructively.

VI. Unintended Consequences

29) The effort required to obtain the 2016 CI financial incentive led me to focus less on non-APP patients in my practice.
30) Efforts to obtain the 2016 CI financial incentive hindered me from providing other essential medical services to my APP patients.
31) The effort required to obtain the 2016 CI financial incentive improved the care of non-APP patients in my practice

ADDITIONAL METHODS FROM PAPER

Pre-Trial Exclusions

Fifty one specialist physicians had no uniquely attributed patients and so contributed no information to the trial.

Data Abstraction

The Advocate data analytics team created extracts from the Cerner EHR registry and billing data for each practice site that contained the data elements necessary to compute the outcome measures for all patients attributed to Trinity physicians. These records were transferred to the University of Pennsylvania, where study staff checked data quality and constructed an analytic data set.

We excluded patients attributed to physicians who did not have at least 1 year of experience as an Advocate network member to allow for adequate historical data.

Bootstrapping for Risk-Standardized Primary Outcome Measures

Only measures for which data were collected in both 2015 and 2016 were included. Within each imputation, we bootstrapped 150 samples from the data, ensuring group balance, and then calculated the mean and standard error for the estimated proportion of evidence-based measures received by each patient.[22] For the RCT, we then used average values for each covariate to compute the risk-standardized value, while for the cohort study we used the 'marginalized approach' in which we assigned every patient to both the treatment and comparison groups and used the difference to estimate the risk-standardized value. Estimates were combined using the standard rules from Rubin.[23]

DEFINITION OF CLINICAL INTEGRATION SCORE

A composite measure, called the clinical integration (CI) score, is a weighted average of measures with an emphasis on chronic disease measures with categories such as coronary artery disease, diabetes care, controlling high blood pressure; population Health measures including screening for cancer, substance use, and depression; and patient satisfaction.

REFERENCES

[1] Burwell, Sylvia M. "Setting value-based payment goals—HHS efforts to improve US health care." New England Journal of Medicine 372.10 (2015): 897-899.

[2] Rosenthal MB, Frank RG, Li Z, and Epstein AM. Early experience with pay-for-performance: From concept to practice. *JAMA*. 2005; 294(14):1788-1793.

[3] Rosenthal MB. Beyond pay for performance—emerging models of provider-payment reform. *N Engl J Med*. 2008; 359(12):1197-1200.

[4] Van Herck, Pieter, et al. "Systematic review: effects, design choices, and context of pay-for-performance in health care." BMC health services research 10.1 (2010): 247.

[5] Eijkenaar, Frank, et al. "Effects of pay for performance in health care: a systematic review of systematic reviews." Health policy 110.2 (2013): 115-130.

[6] Petersen, Laura A., et al. "Effects of individual physician-level and practice-level financial incentives on hypertension care: a randomized trial." JAMA 310.10 (2013): 1042-1050.

[7] Petersen LA, Woodard LD, Urech T, Daw C, Sookanan S. Does pay-for-performance improve the quality of health care? Ann Intern Med. 2006; 145(4):265-272.

[8] Scott A, Sivey P, Ait Ouakrim D, et al. The effect of financial incentives on the quality of health care provided by primary care physicians. Cochrane Database Syst Rev. 2011; 9(9):CD008451.

[9] Houle SK, McAlister FA, Jackevicius CA, Chuck AW, Tsuyuki RT. Does performance-based remuneration for individual health care practitioners affect patient care? a systematic review. Ann Intern Med. 2012; 157(12):889-899.

[10] Thaler RH and Sunstein CR. *Nudge: Improving Decisions about Health, Wealth, and Happiness*. New Haven: Yale University Press, 2008.

[11] Kahneman D. *Thinking, Fast and Slow*. New York: Farrar, Straus and Giroux, 2011.

[12] Volpp KG, Troxel AB, Pauly MV, et al. A randomized controlled trial of financial incentives for smoking cessation. *N Engl J Med*. 2009; 360:699-709.

[13] Volpp KG, John LK, Troxel AB, Norton LA, Fassbender JE, and Loewenstein G. Financial incentive-based approaches for weight loss: A randomized trial. *JAMA*. 2008; 300:2631-2637.

[14] Volpp KG, Loewenstein G, Troxel AB, et al. A test of financial incentives to improve

Warfarin adherence. *BMC Health Serv Res*. 2008; 8:272.

[15] Sutherland K, Christianson JB, and Leatherman S. Impact of targeted financial incentives on personal health behavior: A review of the literature. *Med Care Res Rev*. 2008; 65.6 suppl: 36S-78S.

[16] Giuffrida A and Torgerson DJ. Should we pay the patient? Review of financial incentives to enhance patient compliance. *BMJ*. 1998; 315(7110):703-707.

[17] Kane RL, Johnson PE, Town RJ, and Butler M. A structured review of the effect of economic incentives on consumers' preventive behavior. *Am J Prev Med*. 2004;27(4):327-352.

[18] Stitzer ML, Petry NM, and Peirce J. Motivational incentives research in the National Drug Abuse Treatment Clinical Trials Network. *J Subst Abuse Treat*. 2010; 38(Suppl. 1):S61-69.

[19] Jeffery RW, Gerber WM, Rosenthal BS, Lindquist RA. Monetary Contracts in Weight Control: Effectiveness of Group and Individual Contracts of Varying Size. *J Consult Clin Psychol*. 1983; 51(2):242-248.

[20] Jeffery RW. Financial incentives and weight control. *Prev Med*. 2012; 55:S61-S67.

[21] Angrist, Joshua D., and Jörn-Steffen Pischke. Mostly harmless econometrics: An empiricist's companion. Princeton university press, 2008.

[22] Muller, Clemma J., and Richard F. MacLehose. "Estimating predicted probabilities from logistic regression: different methods correspond to different target populations." International journal of epidemiology 43.3 (2014): 962-970.

[23] Rubin, Donald B. Multiple imputation for nonresponse in surveys. Vol. 81. John Wiley & Sons, 2004.

Supplement 2. Additional Appendix Materials

eFIGURE 1 - Loss Aversion with Larger Bonus Size Arm

EXAMPLE OF PROFORMAS SENT TO PROVIDERS:

SUPPLEMENTAL PRO FORMA for YOUR PRE-FUNDED INCENTIVE ACCOUNT*

The graph below shows the size (in dollars) of your pre-funded 2016 CI Incentive account. Below the graph, you will find the amount of your 2016 CI Incentive that you can access in advance.

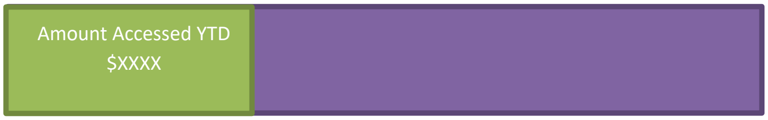

YOUR 2016 CI INCENTIVE ACCOUNT $XXXXX

Eligible pre-funded 2016 CI incentive amount for advanced access: $YYYY

Remaining incentive dollars you may draw out in advance: $ZZZZ

YOUR PROJECTED 2016 CI INCENTIVE BASED ON YTD PERFORMANCE IS:

Jan 2016

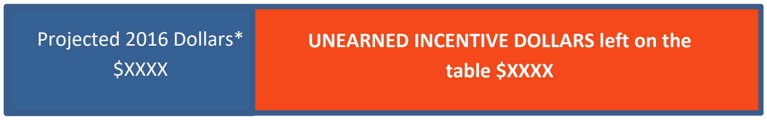

*If you perform the same as last year you will earn this much in 2016 and leave the corresponding amount in red on the table.

Q1

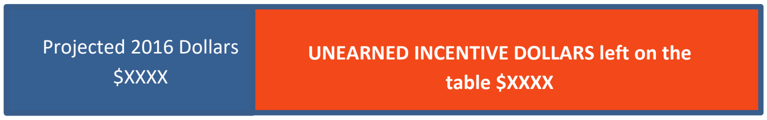

Q2

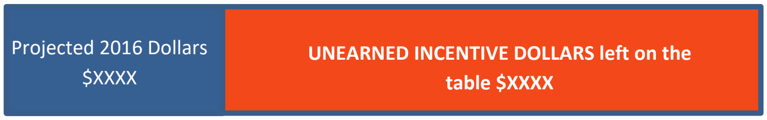

Q3

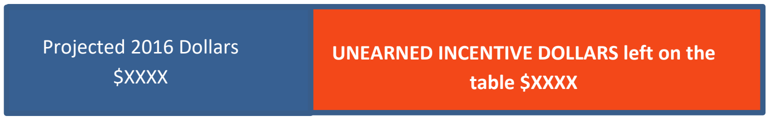

Q4

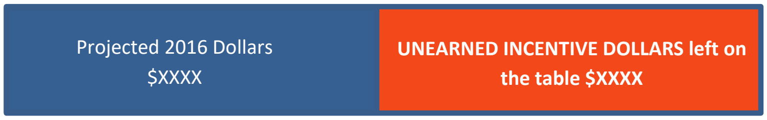

*NOTE: Projections are based on changes in performance holding other aspects equal and are based on latest available data (2014 CI Year Incentive Opportunity amount). Any significant changes in attributed members (for the PCPs) or allowable billings/unique patients (for the Specialists) will impact the actual 2016 CI incentive performance and opportunity, and correspondingly the accuracy of the projections on this Supplemental ProForma.

eFIGURE 2 - Increased Social Pressure with Larger Bonus Size Arm

SUPPLEMENTAL PRO FORMA for ENHANCED GROUP INCENTIVE*

The bar graph below shows the additional incentive dollars you can receive through group performance versus prior years.
- Blue Bar: In 2014, you earned $3,000 of your CI incentive from the PHO pool based on the Trinity PHO score of 79%.
- Red Bar: In the current 2016 year, with the new program design and if your group performs the same as 2014, you would earn $4,590 of your CI incentive based on your group performance.
- Green Bar: In the current 2016 year, with the new program design and if the group performance increases to 90%, you would earn $5,095 of your CI incentive based on your group performance.

That means, in 2016 if your group performs at 90%, you could earn $2,095 more than you did in 2014 based on your group performance.

"Group" refers to the performance of the physicians in Arm 3 Enhanced Group Incentive *only*.

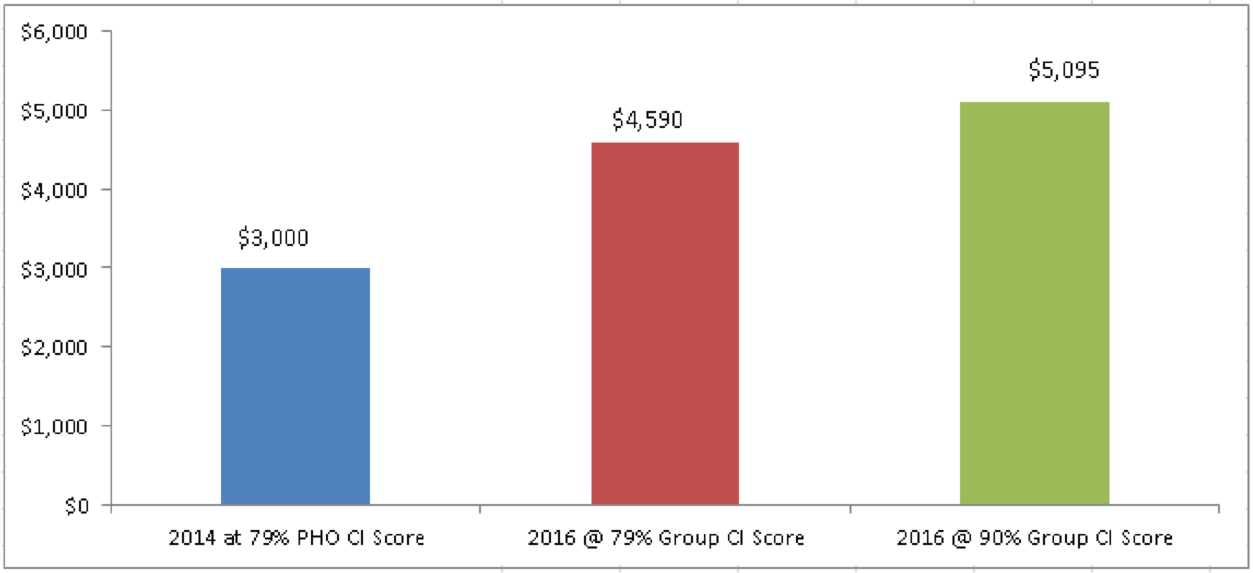

The individual component of your 2016 CI opportunity is decreased by $YYY.

The current Group (Arm 3) performance shows the following metrics that are hurting the Group CI Score:

COMPLETION RATES

Physician Name	Practice Site
1.	
2.	
3.	
4.	
5.	
6.	
7.	
8.	
9.	
10.	

ASTHMA MANAGEMENT

Physician Name	Practice Site
1.	
2.	
3.	
4.	
5.	

COLORECTAL CANCER SCREENING

Physician Name	Practice Site
1.	
2.	
3.	
4.	
5.	

eTABLE 1: Measures in Composite Quality Measure Score for Chronic Disease Patients

Chronic Disease Registry	Advocate Measure Name	Study Measure Name	Measure Definition
Asthma Care	Asthma Action Plan	Asthma Action Plan	Eligible patients 5-64 years of age. A documented action plan containing: a list of medications to take for asthma, instructions regarding how the patient should monitor asthma, and instructions regarding what changes in treatment should result from observed changes in symptoms.
Asthma Care	Asthma Control Treatment Assessed	Asthma Control Treatment Assessed	Eligible patients 5-64 years of age. Control assessment performed and documented in the medical record
Asthma Care	Asthma Medication Management	Asthma Medication Management	Eligible patients 5-64 years of age with asthma. Documentation indicating at least one prescription for an asthma controller medication filled during the measurement period.
Asthma Care, Diabetes Care, Congestive Heart Failure, Chronic Obstructive Pulmonary Disease, Ischemic Vascular Disease/Coronary Artery Disease	Tobacco Use Cessation Counseling	Tobacco Use Cessation Counseling	Patient has tobacco Cessation Counseling and Treatment completed in measurement period.
Asthma Care, Diabetes Care, Congestive Heart Failure, Chronic Obstructive Pulmonary Disease, Ischemic Vascular Disease/Coronary Artery	Tobacco Use Assessment	Tobacco Use Assessment	Patient has documentation of being identified as a Tobacco Non-User or User.

Disease			
Diabetes Care	Percent HbA1c Test	Hemoglobin A1c Testing	Eligible patients ages >=19 and <76. Patient has an HbA1c test performed and resulted during the current measurement period and documented.
Diabetes Care	Percent with A1c result <8	HbA1c Control (<8%)	Eligible patients ages >=19 and <76. Patient has HbA1c test performed and resulted during the current measurement period and documented with the lowest result being less <8%.
Diabetes Care	Percent with A1c result >9	HbA1c Poor Control (>9%)	Eligible patients ages >=19 and <76. Patient has an HbA1c test performed and resulted during the current measurement period with the result being >=9% or patient did not receive test in current measurement period.
Diabetes Care	Annual Eye Exam	Diabetes: Eye Exam Performed	Eligible patients ages >=19 and <76. Patient has a retinal eye exam performed and documented.
Diabetes Care	Nephropathy Monitoring	Diabetes: Medical Attention for Nephropathy	Eligible patients ages >=19 and <76. The patient has a nephropathy screening test performed and reported during the current measurement period or patient has evidence of ACE inhibitor/ARB therapy administration or patient has a documented evidence of Nephropathy.
Diabetes Care, Ischemic Vascular Disease/Coronary Artery Disease	Blood Pressure Control <140/90 mm/Hg	Blood Pressure Control (<140/90 mm/Hg)	Eligible patients >=19 and <76. Patient has blood pressure taken and reported during the current measurement period and documented.
Diabetes Care	Foot Exam	Diabetes: Foot Exam	Eligible patients >=19 and <76. Patient has a foot exam performed and resulted during the measurement period and

			documented.
Diabetes Care, Ischemic Vascular Disease/Coronary Artery Disease	Body Mass Index Assessment	Adult BMI	Eligible patients >=19 and <76. Patient has a Body Mass Index or calculated BMI performed and reported during current measurement period.
Diabetes Care, Congestive Heart Failure, Ischemic Vascular Disease/Coronary Artery Disease	Depression Screening and Follow Up Plan	Depression Screening and Follow Up Plan	Eligible patients >= 18 years. Patient has a depression screening performed during the measurement period. If positive screening, then patient must have a follow up action plan documented.
Congestive Heart Failure	CHF Appropriate Medication Outpatient – Beta Blockers	CHF Appropriate Medication Outpatient – Beta Blockers	Eligible patients >= 19 years. Patient has a beta blocker therapy prescribed during the current measurement period and documented.
Congestive Heart Failure	CHF Appropriate Medication Outpatient – ACEi or ARBs	CHF Appropriate Medication Outpatient – ACEi or ARBs	Eligible patients >=19 years. Patient has an ACEi or ARB medication prescribed during the current measurement period and documented.
Congestive Heart Failure	Documentation of Designated Decision Maker for Medical Care Form	Documentation of Designated Decision Maker for Medical Care Form	Eligible patients >=65 years. Patient has a documented Designated Decision Maker for Medical Care.
Chronic Obstructive Pulmonary Disease	COPD Spirometry Evaluation	COPD Spirometry Evaluation	Eligible patients >=40 years. Patient had a spirometry evaluation performed and documented.
Ischemic Vascular Disease/Coronary Artery Disease	IVD/CAD – Use of Anti-Platelet Medication	IVD/CAD – Use of Anti-Platelet Medication	Eligible patients >=19 years. Patient has documentation of an anti-platelet medication during the measurement year.
Ischemic Vascular Disease/Coronary Artery Disease	IVD/CAD – Blood Pressure Measurement	IVD/CAD – Blood Pressure Measurement	Eligible patients >+19 years. Patient has a systolic blood pressure value taken during the current measurement period and a diastolic blood pressure value from the same date and patient does not have

			an emergency visit or an inpatient visit with the same encounter.

eAPPENDIX 1 - Propensity Matching Methods and Graphs for the Area of Common Support

Propensity matching was performed in a two-step approach because not all physicians had historic trend data. In the first step, we used a logistic model with a dependent variable of participation in the Trinity PHO and independent variables of physician demographics, 2015 (pre-) composite quality score (on measures included in the study), and the trend from 2014-2015. This resulted in a match for 28 of the 33 physicians. The remaining 5 physicians were matched using a similar model without the 2014-2015 trend because these physicians did not have adequate historical data. In total, all 33 physicians in the RCT who received larger bonus sizes were matched to a physician in the no larger bonus size group in a 1:1 match using a 2 digit match.

The area of common support is shown below using kernel density.

eFIGURE 3

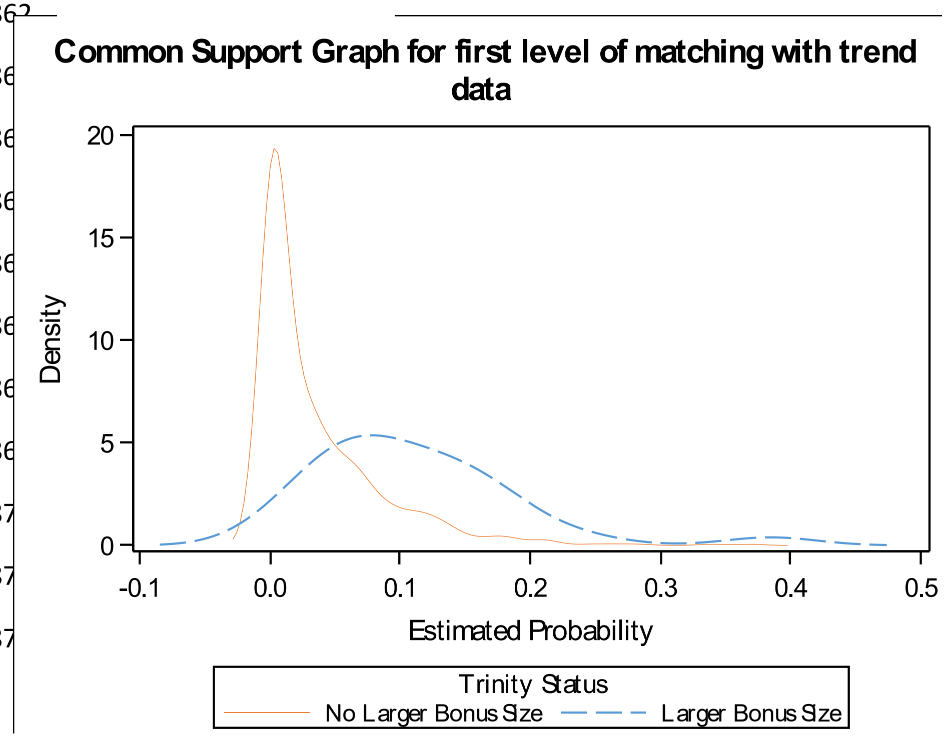

eFIGURE 4

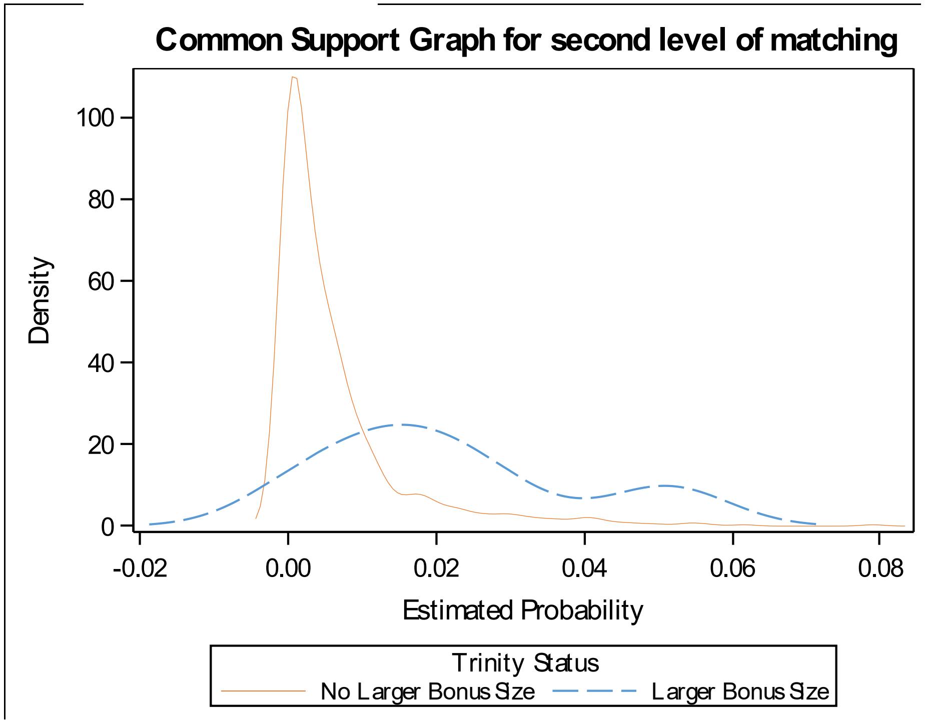

eAPPENDIX 2: Test of Trend Methods

We compared the trend in physician performance for Larger Bonus Size and matched No Larger Bonus Size physicians prior to the 2016 intervention. Eleven measures from the main analysis existed beginning in 2011.[1]

- Diabetes: Eye Exam Performed
- Diabetes: HbA1c Control (<8%)
- Diabetes: HbA1c Poor Control (>9%)
- Diabetes: Hemoglobin A1c Testing
- Diabetes: Medical Attention for Nephropathy
- CHF Appropriate Medication Outpatient – ACEi or ARBs
- CHF Appropriate Medication Outpatient – Beta Blockers
- IVD - Adult BMI
- IVD - Blood Pressure Control (<140/90 mm/Hg)
- IVD– Blood Pressure Measurement
- IVD– Use of Anti-Platelet Medication

We constructed a physician-year performance measure defined as the number of patients meeting evidence-based quality measures divided by the number of patients who should meet the quality measure. Note this definition allows a patient to be double counted if they are relevant for multiple measures. The performance measure was defined using physician level registry data from 2011 and 2012 and patient level registry data from 2014 and 2015.

To test the trend in performance we ran the following linear regression clustering at the physician level and weighting by number of measures (when indicated):

$$y = \alpha_0 + \alpha_1\, LBS + \alpha_2 Year + \alpha_3 LBS\ x\ Year + \epsilon$$

Where year is a continuous variable and trinity indicates whether the physician is in the Larger Bonus Size (LBS) group.[2] Physicians are included only if they are included in the main analysis.

This analysis demonstrated no significant differences in the trend in performance (Year x Trinity interaction term) in the years prior to the intervention.

[1] The Ischemic Vascular disease measures were for a broader set of patients in the main analysis.
[2] Year is centered at 2010 to ease interpretation of the coefficient on Trinity

eTABLE 2. Complete unadjusted results of randomized controlled trial

Study Measure	Increased Social Pressure + Larger Bonus Size					Loss Aversion + Larger Bonus Size				
	# Patients	2015	# Patients	2016	Difference	# Patients	2015	# Patients	2016	Difference
**Overall**	**1496**	**85%**	**1496**	**89%**	**4%**	**1387**	**84%**	**1387**	**88%**	**4%**
**Asthma Action Plan**	92	86%	72	91%	5%	46	78%	42	87%	9%
**Asthma Control Treatment Assessed**	92	84%	72	91%	8%	46	78%	42	86%	8%
**Asthma Medication Management**	53	94%	35	97%	2%	19	95%	20	94%	0%
**Adult BMI**	737	96%	768	95%	-1%	622	98%	669	96%	-2%
**Blood Pressure Control (<140/90 mm/Hg)**	1388	83%	1406	84%	1%	1307	85%	1326	85%	0%
**IVD/CAD – Blood Pressure Measurement**	1228	96%	1290	96%	0%	1252	98%	1259	98%	0%
**COPD Spirometry Evaluation**	239	54%	288	65%	11%	199	72%	221	81%	9%
**Diabetes: Eye Exam Performed**	586	62%	608	68%	5%	416	55%	430	64%	9%
**Diabetes: Foot Exam**	585	74%	608	89%	15%	416	88%	430	87%	-1%
**HbA1c Control (<8%)**	586	69%	608	72%	4%	416	61%	430	66%	5%
**HbA1c Poor Control (>9%)**	586	77%	608	82%	5%	416	73%	430	76%	3%
**Hemoglobin A1c Testing**	586	96%	608	94%	-2%	416	94%	430	94%	0%
**Diabetes: Medical Attention for Nephropathy**	585	96%	608	96%	0%	416	97%	430	97%	-1%
**CHF Appropriate Medication Outpatient – ACEi or ARBs**	90	80%	64	92%	12%	88	90%	75	91%	1%
**CHF Appropriate Medication Outpatient – Beta Blockers**	26	54%	18	100%	46%	28	89%	23	93%	4%
**IVD/CAD – Use of Anti-Platelet Medication**	198	80%	220	91%	11%	242	90%	273	91%	2%
**Depression Screening and Follow Up Plan**	1233	92%	1233	99%	6%	1172	97%	1172	99%	2%
**Documentation of Designated Decision Maker for Medical Care Form**	539	37%	584	72%	36%	672	10%	682	42%	32%
**Tobacco Use Cessation Counseling**	334	87%	317	92%	5%	352	80%	269	89%	9%
**Tobacco Use Assessment**	1486	97%	1487	99%	1%	1384	98%	1384	99%	1%

Abbreviations**:** BMI, Body Mass Index; IVD, Ischemic Vascular Disease; CAD, Coronary Artery Disease; COPD, Chronic Obstructive Pulmonary Disease; HbA1c, Hemoglobin A1c; CHF, Congestive Heart Failure; ACEi, Angiotensin-converting enzyme (ACE) inhibitor; ARBs, Angiotensin II receptor blockers

Larger Bonus Size Only					Adjusted Pair-Wise Comparison[a]		
# Patients	2015	# Patients	2016	Difference	ISP vs LA 2016 vs 2015	ISP vs AC 2016 vs 2015	LA vs AC 2016 vs 2015
864	88%	864	92%	4%			
55	95%	52	94%	-1%	>0.99	>0.99	>0.99
55	93%	52	93%	0%	>0.99	>0.99	>0.99
23	100%	21	100%	0%	>0.99	>0.99	>0.99
316	92%	359	96%	4%	>0.99	>0.99	0.73
671	84%	730	89%	5%	>0.99	>0.99	>0.99
608	95%	667	98%	3%	>0.99	0.32	0.03 [a]
248	81%	265	87%	6%	>0.99	>0.99	>0.99
231	69%	261	76%	7%	>0.99	>0.99	>0.99
231	85%	261	88%	3%	0.91	>0.99	>0.99
231	58%	261	71%	12%	>0.99	>0.99	>0.99
231	70%	261	80%	10%	>0.99	>0.99	>0.99
231	89%	261	93%	4%	>0.99	>0.99	>0.99
231	97%	261	97%	0%	>0.99	>0.99	>0.99
35	91%	49	91%	0%	>0.99	>0.99	>0.99
12	83%	13	98%	15%	0.15	>0.99	>0.99
107	93%	111	94%	1%	>0.99	>0.99	0.98
622	95%	665	99%	3%	0.80	>0.99	>0.99
296	54%	344	79%	24%	>0.99	>0.99	>0.99
163	90%	179	93%	3%	>0.99	>0.99	>0.99
845	98%	845	98%	0%	>0.99	>0.99	>0.99

[a] Reported p-values for pairwise comparisons of the primary outcome of change in proportion of applicable chronic disease and preventive evidence-based measures meeting or exceeding benchmarks at the patient level use the Holm-Bonferroni correction. Multiple imputation was used for the approximately 11% of participants missing follow-up quality metric scores.

eTABLE 3. Complete unadjusted results of cohort study

Study Measure	Larger Bonus Size					No Larger Bonus Size (comparison group)					Adjusted Pair-Wise Comparison
	**N**	**2015**	**N**	**2016**	**Difference**	**N**	**2015**	**N**	**2016**	**Difference**	**Adjusted P-Value[a]**
**Overall**	**3747**	**85%**	**3747**	**89%**	**4%**	**4371**	**86%**	**4371**	**88%**	**2%**	
**Asthma Action Plan**	193	87%	166	91%	4%	164	84%	128	88%	4%	>0.99
**Asthma Control Treatment Assessed**	193	85%	166	90%	5%	164	81%	129	88%	7%	0.95
**Asthma Medication Management**	95	96%	76	97%	1%	104	93%	74	100%	7%	>0.99
**Adult BMI**	1675	96%	1796	96%	0%	2119	97%	2168	95%	-2%	0.12
**Blood Pressure Control (<140/90 mm/Hg)**	3366	84%	3462	86%	2%	4086	89%	4114	84%	-4%	0.00
**IVD/CAD – Blood Pressure Measurement**	3088	97%	3216	97%	0%	3820	98%	3891	97%	-1%	0.16
**COPD Spirometry Evaluation**	686	69%	774	77%	8%	745	69%	855	72%	3%	0.08
**Diabetes: Eye Exam Performed**	1233	61%	1299	68%	7%	1235	64%	1218	66%	2%	0.16
**Diabetes: Foot Exam**	1232	81%	1299	88%	8%	1235	81%	1218	82%	0%	0.00
**HbA1c Control (<8%)**	1233	64%	1299	70%	6%	1235	72%	1219	71%	0%	0.08
**HbA1c Poor Control (>9%)**	1233	74%	1299	80%	5%	1235	81%	1219	81%	0%	0.09
**Hemoglobin A1c Testing**	1233	94%	1299	94%	0%	1235	95%	1219	94%	-1%	>0.99
**Diabetes: Medical Attention for Nephropathy**	1232	97%	1299	97%	0%	1235	96%	1219	96%	0%	>0.99
**CHF Appropriate Medication Outpatient – ACEi or ARBs**	213	86%	188	91%	5%	261	86%	205	91%	5%	>0.99
**CHF Appropriate Medication Outpatient – Beta Blockers**	66	74%	54	97%	22%	80	91%	75	93%	2%	0.70
**IVD/CAD – Use of Anti-Platelet Medication**	547	87%	604	91%	5%	1061	89%	1118	91%	2%	>0.99
**Depression Screening and Follow Up Plan**	3027	95%	3070	99%	4%	3565	93%	3559	98%	5%	>0.99
**Documentation of Designated Decision Maker for Medical Care Form**	1507	29%	1610	61%	33%	2060	27%	2162	54%	28%	0.17
**Tobacco Use Cessation Counseling**	849	85%	765	91%	6%	698	92%	669	91%	-1%	0.04
**Tobacco Use Assessment**	3715	98%	3716	99%	1%	4341	99%	4343	99%	0%	>0.99

[a] Reported p-values for pairwise comparisons of the primary outcome of change in proportion of applicable chronic disease and preventive evidence-based measures meeting or exceeding benchmarks at the patient level use the Holm-Bonferroni correction. Multiple imputation was used for the approximately 11% of participants missing follow-up quality metric scores. Abbreviations**:** BMI, Body Mass Index; IVD, Ischemic Vascular Disease; CAD, Coronary Artery Disease; COPD, Chronic Obstructive Pulmonary Disease; HbA1c, Hemoglobin A1c; CHF, Congestive Heart Failure; ACEi, Angiotensin-converting enzyme (ACE) inhibitor; ARBs, Angiotensin II receptor blockers

eTABLE 4. Sample Characteristics of Cohort Study for Larger Bonus size without Matching

	Larger Bonus Size	All No Larger Bonus Size	P-value
**Number of physicians**	N = 33	N = 801	
**Age (year), mean (SD)**	57 (10)	53 (10)	0.04
**Tenure (year), mean (SD)**	12 (8)	9 (7)	0.03
**Average No. of APP patients in panel, median (IQR)**	67 (138)	34 (131)	0.06
**Gender, No. (%)**			
Female	15 (45%)	285 (36%)	0.25
Male	18 (55%)	516 (64%)	
**Specialty, No. (%)[a]**			0.00
Family Medicine	14 (42%)	153 (19%)	
Internal Medicine	13 (39%)	214 (27%)	
Pediatrics	4 (12%)	183 (23%)	
Others	2 (6%)	251 (31%)	
**Average No. of Chronic Disease, mean (SD)**	1.60 (0.34)	1.47 (0.38)	0.05
**Number of patients**	N = 3747	N = 70818	
**Age (year), median (IQR)**	64 (18)	68 (18)	<.0001
**Gender, No. (%)**			
Female	2384 (64%)	36880 (52%)	<.0001
Male	1358 (36%)	33758 (48%)	
**Race, No. (%)**			
Black or African American	2667 (71%)	7461 (11%)	<.0001
Caucasian or White	368 (10%)	48658 (69%)	
Other	149 (4%)	4547 (6%)	
Unknown	563 (15%)	10152 (14%)	
**Average No. of Chronic Disease, mean (SD)**	1.6 (0.82)	1.63 (0.83)	0.06

[a]Other physicians includes 1 Cardiologist and 1 Pulmonologist in the Larger Bonus Size cohort. For No Larger Bonus Size cohort, Other physicians includes 28 Allergists/Immunologists, 5 Cardiac Electrophysiologists, 98 Cardiologists, 25 Endocrinologists, 10 Interventional Cardiologists, 6 Pediatric Allergists/Immunologists, 79 Pulmonologists

Abbreviations: SD, standard deviation; IQR, interquartile range.

eFIGURE 5 – Sensitivity Analysis for RCT without Physician Fixed Effect Clustering at Group Practice Level

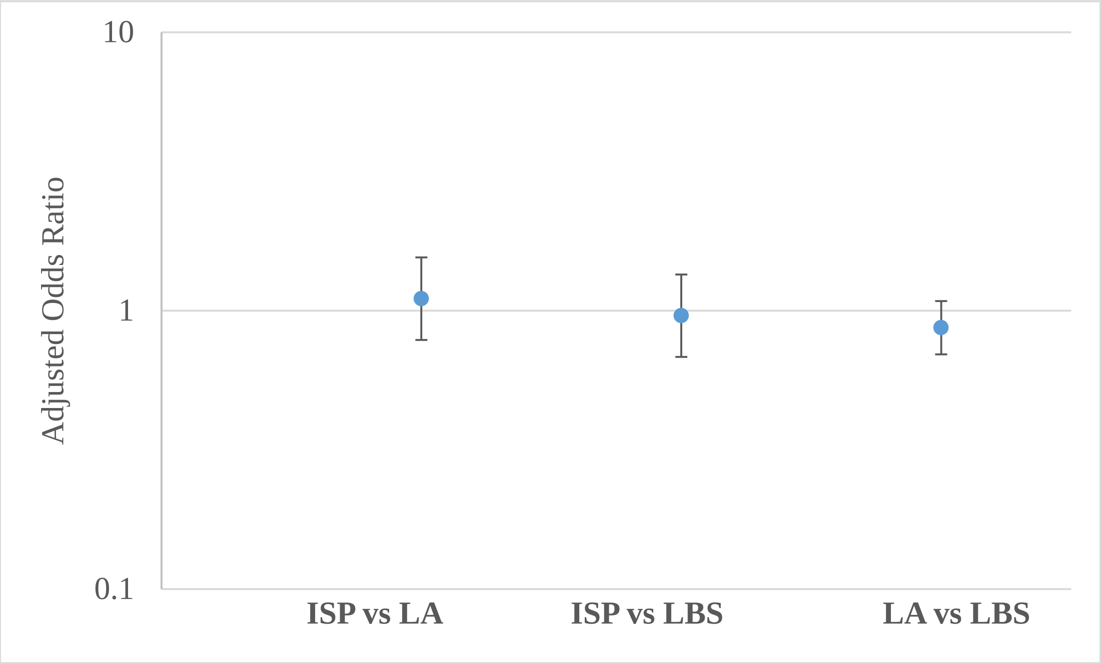

Error bars indicate 95% confidence Intervals

ISP: Larger bonus size + Increased social pressure
LA: Larger bonus size + Loss aversion
LBS: Larger bonus size only (comparison group)

eFIGURE 6 – Sensitivity Analysis for RCT without Imputation (using Complete Case Data)

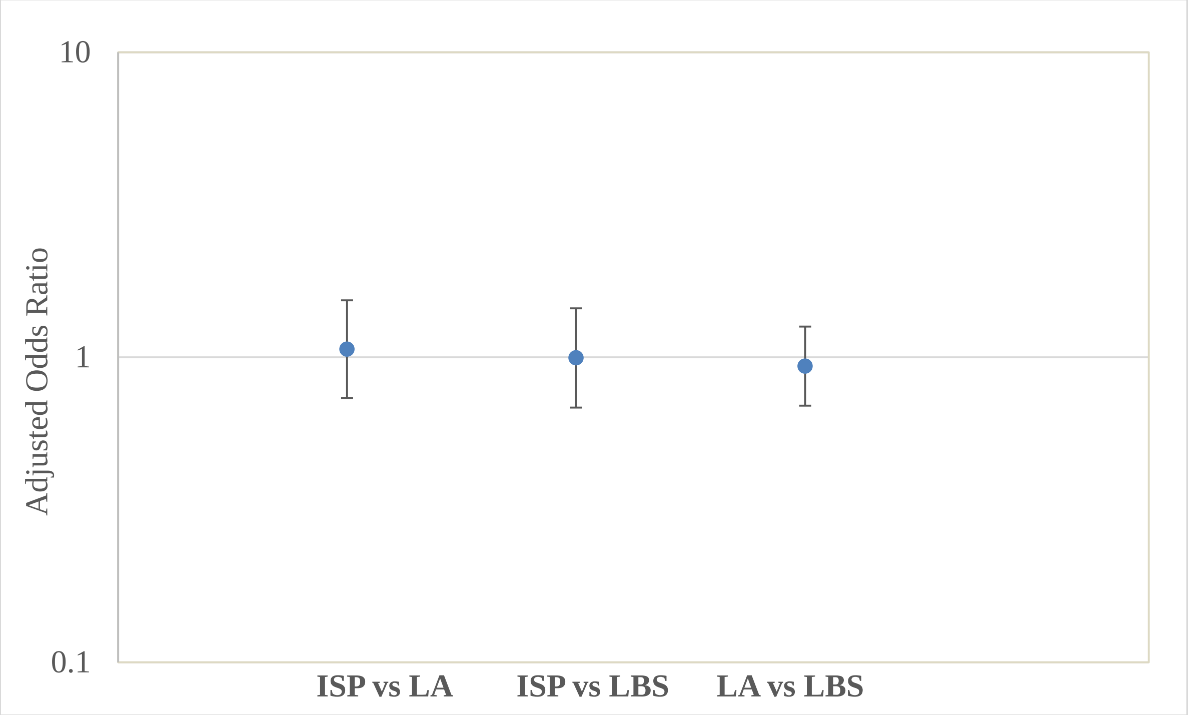

Error bars indicate 95% confidence Intervals

ISP: Larger bonus size + Increased social pressure
LA: Larger bonus size + Loss aversion
LBS: Larger bonus size only (comparison group)

eFIGURE 7 – Sensitivity Analysis for RCT with Physician Random Effect

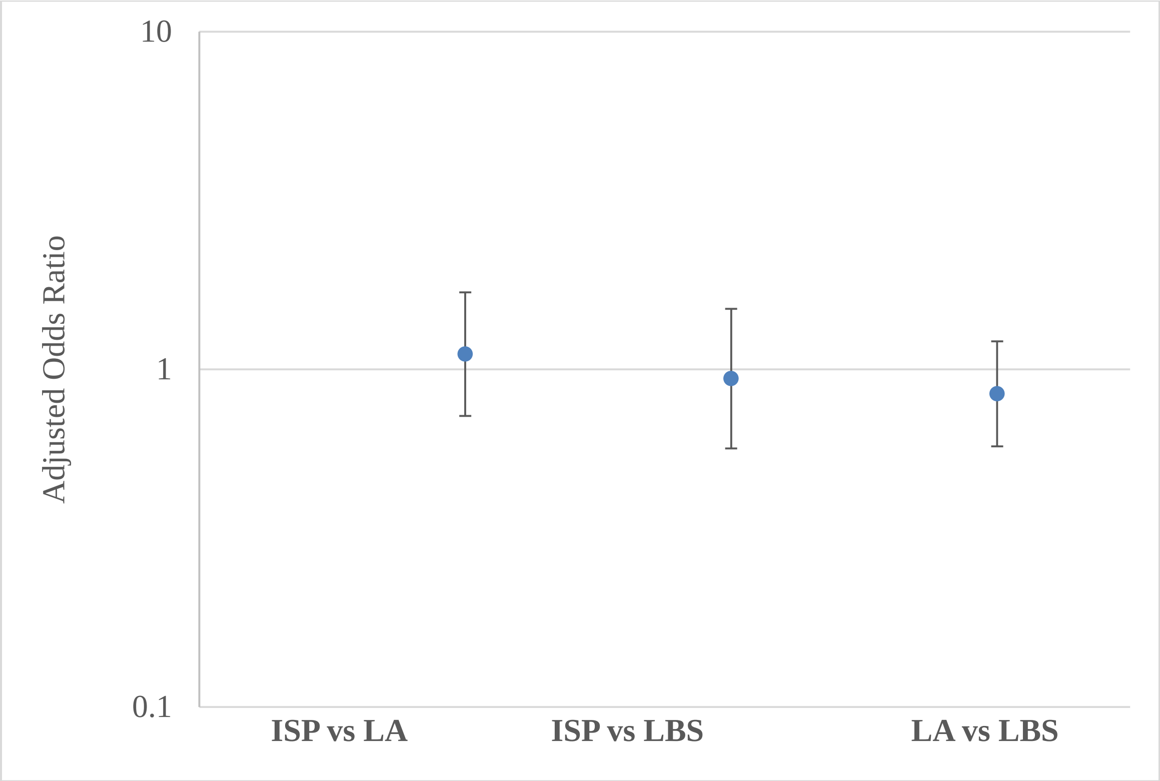

Error bars indicate 95% confidence Intervals

ISP: Larger bonus size + Increased social pressure
LA: Larger bonus size + Loss aversion
LBS: Larger bonus size only (comparison group)

eFIGURE 8 – Sensitivity Analysis of Cohort Study without Imputation (using Complete Case Data)

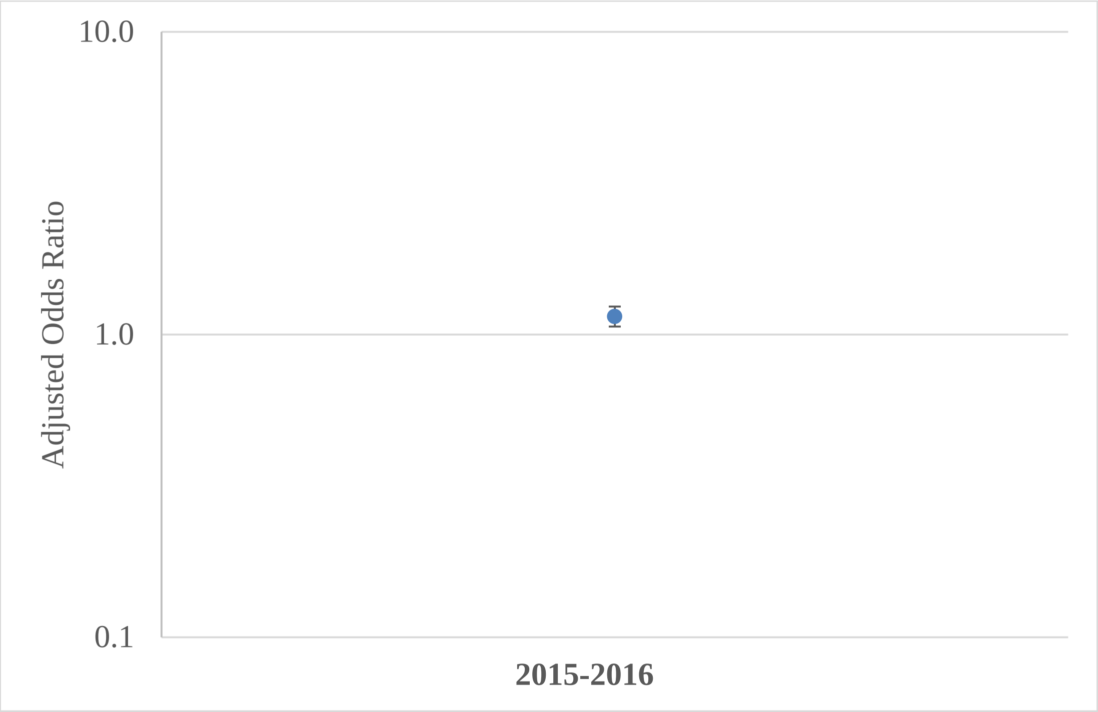

The estimate is the effect of the association between larger bonus size and higher achievement of evidence-based quality measures. The error bars indicate 95% confidence intervals.

eFIGURE 9 – Sensitivity Analysis of Cohort Study without Physician Fixed Effects

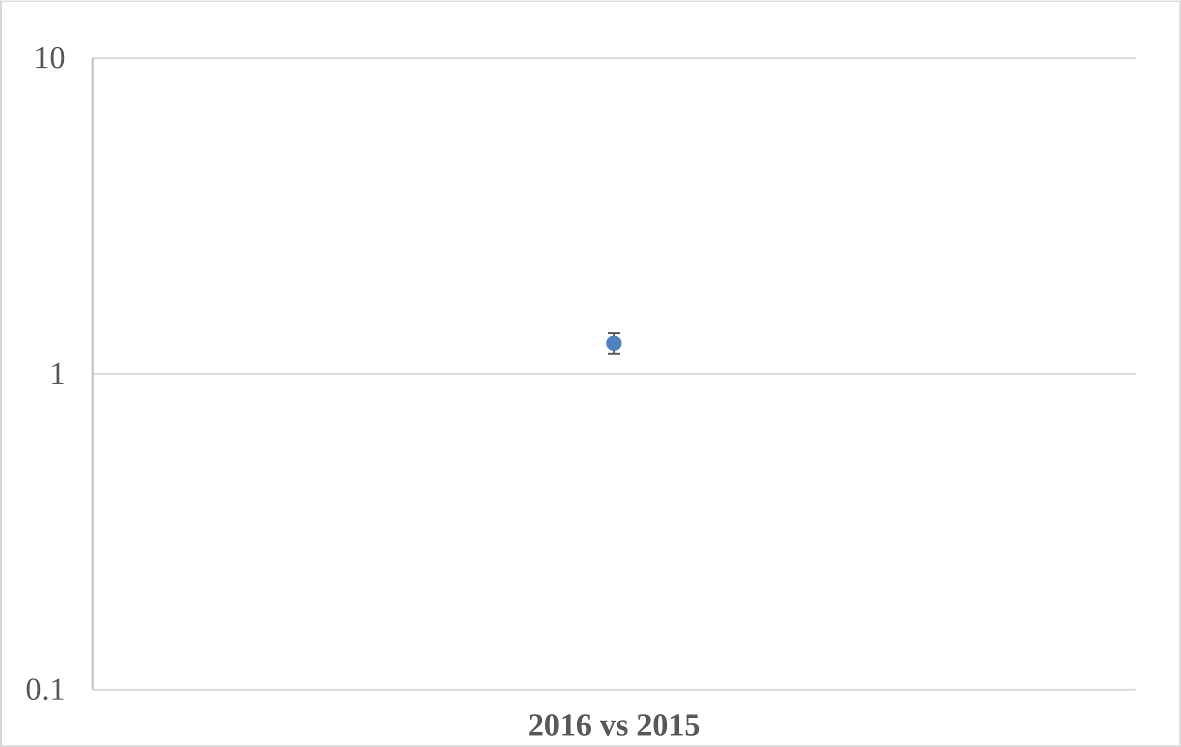

The estimate is the effect of the association between larger bonus size and higher achievement of evidence-based quality measures. The error bars indicate 95% confidence intervals.

eTABLE 5 – Test of Trends for Difference-in-Differences Model Results

Coefficient (SE)	All Physicians, Weighted	Stable Set of Physicians, Weighted
**Year**	-0.007 (0.005)	-0.006 (0.004)
**Trinity**	-0.013 (0.031)	-0.009 (0.030)
**Year x Trinity**	-0.011 (0.008)	-0.012 (0.007)
**Constant**	0.854*** (0.020)	0.851*** (0.019)
**Observations**	186	165
**R^2**	0.116	0.112
**Unique Trinity MDs**	32	18
**Unique Non-Trinity MDs**	33	23

Standard errors in parentheses; * $p < 0.05$, ** $p < 0.01$, *** $p < 0.001$

eTABLE 6: Results of Physician Survey Administered Pre and Post Intervention

	Larger Bonus Size				Loss Aversion & Larger Bonus Size				Increased Social Pressure & Larger Bonus Size			
**Overall**	**Pre**	**Post**	**Change**	**t-test**	**Pre**	**Post**	**Change**	**t-test**	**Pre**	**Post**	**Change**	**t-test**
	**n=24**	**n=14**			**n=26**	**n=13**			**n=21**	**n=7**		
**Baseline Attitudes**	4.21	4.18	-0.04	0.47	3.64	3.69	0.06	0.45	3.98	4.02	0.04	0.44
**Teamwork**	3.89	3.91	0.03	0.48	4.11	3.93	-0.18	0.30	4.18	3.82	-0.37	0.02
**Financial Salience**	3.61	3.36	-0.25	0.33	3.03	3.69	0.67	0.04	3.35	3.35	0.01	0.41
**Practice Environment**	3.69	3.57	-0.12	0.37	4.00	3.80	-0.20	0.04	3.35	3.35	0.01	0.41
**Awareness/Understanding**	3.54	3.77	0.23	0.32	3.67	3.67	0.00	0.50	3.40	3.37	-0.03	0.45
**Individual Impact on Clinical Behavior**	3.48	3.57	0.10	0.43	3.37	3.22	-0.15	0.26	3.47	3.46	-0.01	0.48
**Unintended Consequences**	2.83	3.10	0.27	0.14	2.85	3.33	0.48	0.01	3.14	3.25	0.11	0.25
