# Supplementary material for: Effect of Financial Bonus Size, Loss Aversion, and Increased Social Pressure on Physician Pay-for-Performance: A Randomized Clinical Trial and Cohort Study
Source: JAMA Netw Open. 2019 Feb 8;2(2):e187950. doi: 10.1001/jamanetworkopen.2018.7950 (PMC6484616; doi:10.1001/jamanetworkopen.2018.7950)
Supplement: Supplement 2. — eFigure 1. Loss Aversion With Larger Bonus Size Arm eFigure 2. Increased Social Pressure With Larger Bonus Size Arm eFigure 3. Common Support Graph for First Level of Matching With Trend Data eFigure 4. Common Support Graph for Second Level of Matching eFigure 5. Sensitivity Analysis for RCT Without Physician Fixed Effect Clustering at Group Practice Level eFigure 6. Sensitivity Analysis for RCT Without Imputation (Using Complete Case Data) eFigure 7. Sensitivity Analysis for RCT With Physician Random Effect eFigure 8. Sensitivity Analysis of Cohort Study Without Imputation (Using Complete Case Data) eFigure 9. Sensitivity Analysis of Cohort Study Without Physician Fixed Effects eTable 1. Measures in Composite Quality Measure Score for Chronic Disease Patients eTable 2. Complete Unadjusted Results of Randomized Clinical Trial eTable 3. Complete Unadjusted Results of Cohort Study eTable 4. Sample Characteristics of Cohort Study for Larger Bonus Size Without Matching eTable 5. Test of Trends for Difference-in-Differences Model Results eTable 6. Results of Physician Survey Administered Before and After Intervention eMethods 1. Propensity Matching Methods and Graphs for the Area of Common Support eMethods 2. Test of Trend Methods [file jamanetwopen-2-e187950-s002.pdf]

Supplementary Online Content

Navathe AS, Volpp KG, Caldarella KL, et al. Effect of financial bonus size, loss aversion, and increased social pressure on physician pay-for-performance: a randomized clinical trial and cohort study. *JAMA Netw Open*. 2019;2(2):e187950. doi:10.1001/jamanetworkopen.2018.7950

eFigure 1. Loss Aversion with Larger Bonus Size Arm

eFigure 2. Increased Social Pressure With Larger Bonus Size Arm

eFigure 3. Common Support Graph for First Level of Matching With Trend Data

eFigure 4. Common Support Graph for Second Level of Matching

eFigure 5. Sensitivity Analysis for RCT Without Physician Fixed Effect Clustering at Group Practice Level

eFigure 6. Sensitivity Analysis for RCT Without Imputation (Using Complete Case Data)

eFigure 7. Sensitivity Analysis for RCT With Physician Random Effect

eFigure 8. Sensitivity Analysis of Cohort Study Without Imputation (Using Complete Case Data)

eFigure 9. Sensitivity Analysis of Cohort Study Without Physician Fixed Effects

eTable 1. Measures in Composite Quality Measure Score for Chronic Disease Patients

eTable 2. Complete Unadjusted Results of Randomized Clinical Trial

eTable 3. Complete Unadjusted Results of Cohort Study

eTable 4. Sample Characteristics of Cohort Study for Larger Bonus Size Without Matching

eTable 5. Test of Trends for Difference-in-Differences Model Results

eTable 6. Results of Physician Survey Administered Before and After Intervention

eMethods 1. Propensity Matching Methods and Graphs for the Area of Common Support

eMethods 2. Test of Trend Methods

This supplementary material has been provided by the authors to give readers additional information about their work.

© 2019 Navathe AS et al. *JAMA Network Open*.

Supplement 2. Additional Appendix Materials

eFigure 1. Loss Aversion with Larger Bonus Size Arm

EXAMPLE OF PRO FORMAS SENT TO PROVIDERS:

SUPPLEMENTAL PRO FORMA for YOUR PRE-FUNDED INCENTIVE ACCOUNT*

The graph below shows the size (in dollars) of your pre-funded 2016 CI Incentive account. Below the graph, you will find the amount of your 2016 CI Incentive that you can access in advance.

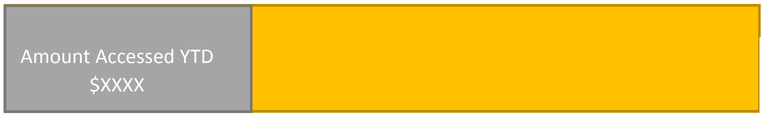

YOUR 2016 CI INCENTIVE ACCOUNT $XXXXX

Eligible pre-funded 2016 CI incentive amount for advanced access: $YYYY

Remaining incentive dollars you may draw out in advance: $ZZZZ

YOUR PROJECTED 2016 CI INCENTIVE BASED ON YTD PERFORMANCE IS:

Jan 2016

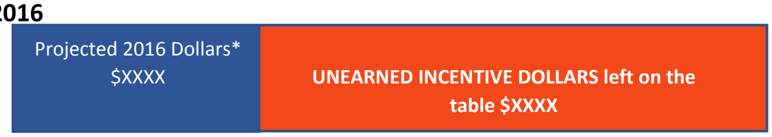

*If you perform the same as last year you will earn this much in 2016 and leave the corresponding amount in red on the table.

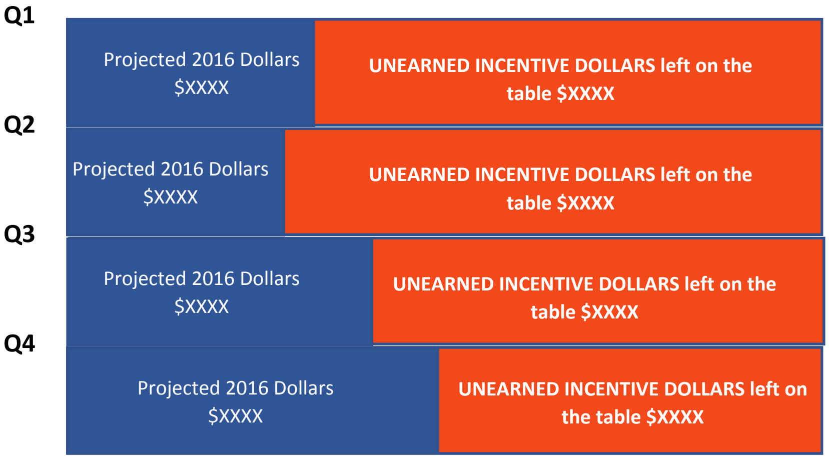

*NOTE: Projections are based on changes in performance holding other aspects equal and are based on latest available data (2014 CI Year Incentive Opportunity amount). Any significant changes in attributed members (for the PCPs) or allowable billings/unique patients (for the Specialists) will impact the actual 2016 CI incentive performance and opportunity, and correspondingly the accuracy of the projections on this Supplemental ProForma.

© 2019 Navathe AS et al. *JAMA Network Open*.

eFigure 2. Increased Social Pressure With Larger Bonus Size Arm

SUPPLEMENTAL PRO FORMA for ENHANCED GROUP INCENTIVE*

The bar graph below shows the additional incentive dollars you can receive through group performance versus prior years.
- Blue Bar: In 2014, you earned $3,000 of your CI incentive from the PHO pool based on the Trinity PHO score of 79%.
- Red Bar: In the current 2016 year, with the new program design and if your group performs the same as 2014, you would earn $4,590 of your CI incentive based on your group performance.
- Green Bar: In the current 2016 year, with the new program design and if the group performance increases to 90%, you would earn $5,095 of your CI incentive based on your group performance.

That means, in 2016 if your group performs at 90%, you could earn $2,095 more than you did in 2014 based on your group performance.
"Group" refers to the performance of the physicians in Arm 3 Enhanced Group Incentive *only*.

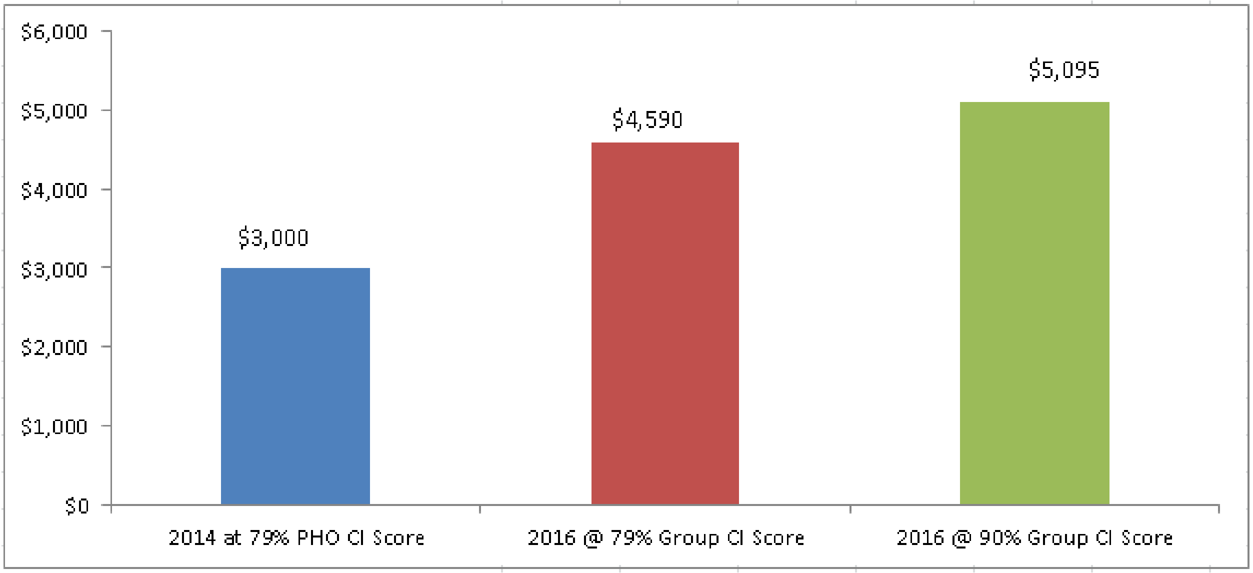

The individual component of your 2016 CI opportunity is decreased by $YYY.

The current Group (Arm 3) performance shows the following metrics that are hurting the Group CI Score:

COMPLETION RATES

Physician Name	Practice Site
1.	
2.	
3.	
4.	
5.	
6.	
7.	
8.	
9.	
10.	

ASTHMA MANAGEMENT

Physician Name	Practice Site
1.	
2.	
3.	
4.	
5.	

COLORECTAL CANCER SCREENING

Physician Name	Practice Site
1.	
2.	
3.	
4.	
5.	

© 2019 Navathe AS et al. *JAMA Network Open*.

eTable 1. Measures in Composite Quality Measure Score for Chronic Disease Patients

Chronic Disease Registry	Advocate Measure Name	Study Measure Name	Measure Definition
Asthma Care	Asthma Action Plan	Asthma Action Plan	Eligible patients 5-64 years of age. A documented action plan containing: a list of medications to take for asthma, instructions regarding how the patient should monitor asthma, and instructions regarding what changes in treatment should result from observed changes in symptoms.
Asthma Care	Asthma Control Treatment Assessed	Asthma Control Treatment Assessed	Eligible patients 5-64 years of age. Control assessment performed and documented in the medical record
Asthma Care	Asthma Medication Management	Asthma Medication Management	Eligible patients 5-64 years of age with asthma. Documentation indicating at least one prescription for an asthma controller medication filled during the measurement period.
Asthma Care, Diabetes Care, Congestive Heart Failure, Chronic Obstructive Pulmonary Disease, Ischemic Vascular Disease/Coronary Artery Disease	Tobacco Use Cessation Counseling	Tobacco Use Cessation Counseling	Patient has tobacco Cessation Counseling and Treatment completed in measurement period.
Asthma Care, Diabetes Care, Congestive Heart Failure, Chronic Obstructive Pulmonary Disease, Ischemic	Tobacco Use Assessment	Tobacco Use Assessment	Patient has documentation of being identified as a Tobacco Non-User or User.

© 2019 Navathe AS et al. *JAMA Network Open*.

Vascular Disease/Coronary Artery Disease			
Diabetes Care	Percent HbA1c Test	Hemoglobin A1c Testing	Eligible patients ages >=19 and <76. Patient has an HbA1c test performed and resulted during the current measurement period and documented.
Diabetes Care	Percent with A1c result <8	HbA1c Control (<8%)	Eligible patients ages >=19 and <76. Patient has HbA1c test performed and resulted during the current measurement period and documented with the lowest result being less <8%.
Diabetes Care	Percent with A1c result >9	HbA1c Poor Control (>9%)	Eligible patients ages >=19 and <76. Patient has an HbA1c test performed and resulted during the current measurement period with the result being >=9% or patient did not receive test in current measurement period.
Diabetes Care	Annual Eye Exam	Diabetes: Eye Exam Performed	Eligible patients ages >=19 and <76. Patient has a retinal eye exam performed and documented.
Diabetes Care	Nephropathy Monitoring	Diabetes: Medical Attention for Nephropathy	Eligible patients ages >=19 and <76. The patient has a nephropathy screening test performed and reported during the current measurement period or patient has evidence of ACE inhibitor/ARB therapy administration or patient has a documented evidence of Nephropathy.

© 2019 Navathe AS et al. *JAMA Network Open*.

Diabetes Care, Ischemic Vascular Disease/Coronary Artery Disease	Blood Pressure Control <140/90 mm/Hg	Blood Pressure Control (<140/90 mm/Hg)	Eligible patients >=19 and <76. Patient has blood pressure taken and reported during the current measurement period and documented.
Diabetes Care	Foot Exam	Diabetes: Foot Exam	Eligible patients >=19 and <76. Patient has a foot exam performed and resulted during the measurement period and documented.
Diabetes Care, Ischemic Vascular Disease/Coronary Artery Disease	Body Mass Index Assessment	Adult BMI	Eligible patients >=19 and <76. Patient has a Body Mass Index or calculated BMI performed and reported during current measurement period.
Diabetes Care, Congestive Heart Failure, Ischemic Vascular Disease/Coronary Artery Disease	Depression Screening and Follow Up Plan	Depression Screening and Follow Up Plan	Eligible patients >= 18 years. Patient has a depression screening performed during the measurement period. If positive screening, then patient must have a follow up action plan documented.
Congestive Heart Failure	CHF Appropriate Medication Outpatient – Beta Blockers	CHF Appropriate Medication Outpatient – Beta Blockers	Eligible patients >= 19 years. Patient has a beta blocker therapy prescribed during the current measurement period and documented.
Congestive Heart Failure	CHF Appropriate Medication Outpatient – ACEi or ARBs	CHF Appropriate Medication Outpatient – ACEi or ARBs	Eligible patients >=19 years. Patient has an ACEi or ARB medication prescribed during the current measurement period and documented.
Congestive Heart Failure	Documentation of Designated Decision Maker for Medical Care Form	Documentation of Designated Decision Maker for Medical Care Form	Eligible patients >=65 years. Patient has a documented Designated Decision Maker for Medical Care.

© 2019 Navathe AS et al. *JAMA Network Open*.

Chronic Obstructive Pulmonary Disease	COPD Spirometry Evaluation	COPD Spirometry Evaluation	Eligible patients >=40 years. Patient had a spirometry evaluation performed and documented.
Ischemic Vascular Disease/Coronary Artery Disease	IVD/CAD – Use of Anti-Platelet Medication	IVD/CAD – Use of Anti-Platelet Medication	Eligible patients >=19 years. Patient has documentation of an anti-platelet medication during the measurement year.
Ischemic Vascular Disease/Coronary Artery Disease	IVD/CAD – Blood Pressure Measurement	IVD/CAD – Blood Pressure Measurement	Eligible patients >+19 years. Patient has a systolic blood pressure value taken during the current measurement period and a diastolic blood pressure value from the same date and patient does not have an emergency visit or an inpatient visit with the same encounter.

© 2019 Navathe AS et al. *JAMA Network Open*.

eMethods 1. Propensity Matching Methods and Graphs for the Area of Common Support

Propensity matching was performed in a two-step approach because not all physicians had historic trend data. In the first step, we used a logistic model with a dependent variable of participation in the Trinity PHO and independent variables of physician demographics, 2015 (pre-) composite quality score (on measures included in the study), and the trend from 2014-2015. This resulted in a match for 28 of the 33 physicians. The remaining 5 physicians were matched using a similar model without the 2014-2015 trend because these physicians did not have adequate historical data. In total, all 33 physicians in the RCT who received larger bonus sizes were matched to a physician in the no larger bonus size group in a 1:1 match using a 2 digit match.

The area of common support is shown below using kernel density.

eFigure 3.

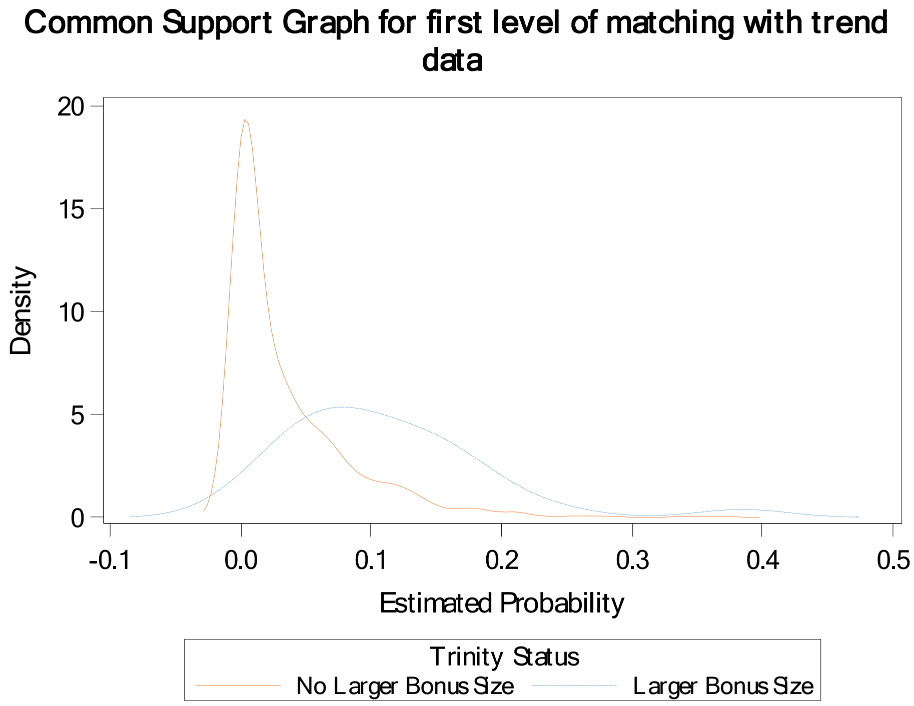

eFigure 4.

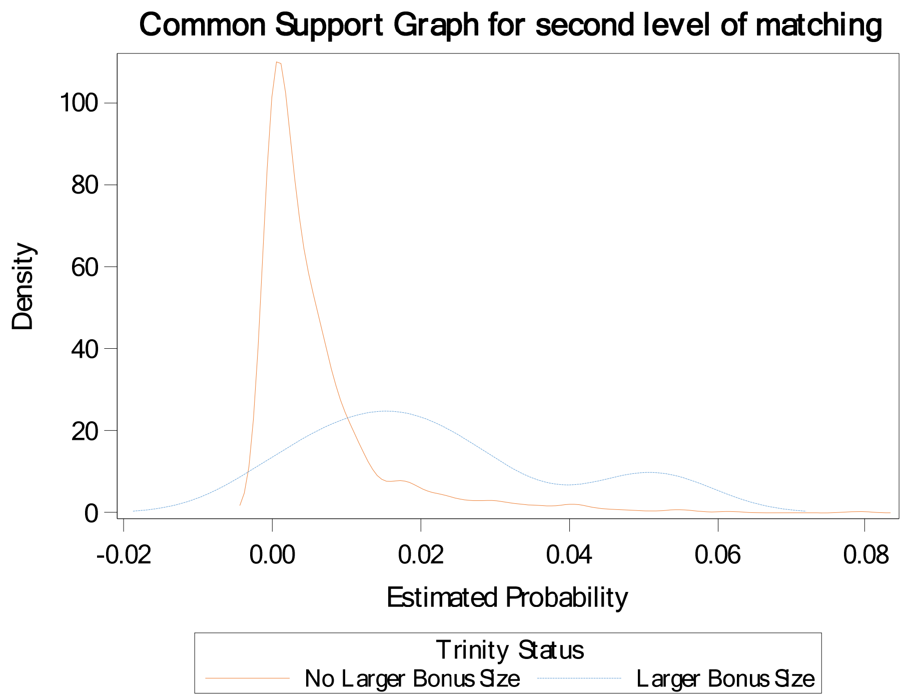

© 2019 Navathe AS et al. *JAMA Network Open*.

eMethods 2. Test of Trend Methods

We compared the trend in physician performance for Larger Bonus Size and matched No Larger Bonus Size physicians prior to the 2016 intervention. Eleven measures from the main analysis existed beginning in 2011.[1]

- Diabetes: Eye Exam Performed
- Diabetes: HbA1c Control (<8%)
- Diabetes: HbA1c Poor Control (>9%)
- Diabetes: Hemoglobin A1c Testing
- Diabetes: Medical Attention for Nephropathy
- CHF Appropriate Medication Outpatient – ACEi or ARBs
- CHF Appropriate Medication Outpatient – Beta Blockers
- IVD - Adult BMI
- IVD - Blood Pressure Control (<140/90 mm/Hg)
- IVD– Blood Pressure Measurement
- IVD– Use of Anti-Platelet Medication

We constructed a physician-year performance measure defined as the number of patients meeting evidence-based quality measures divided by the number of patients who should meet the quality measure. Note this definition allows a patient to be double counted if they are relevant for multiple measures. The performance measure was defined using physician level registry data from 2011 and 2012 and patient level registry data from 2014 and 2015.

To test the trend in performance we ran the following linear regression clustering at the physician level and weighting by number of measures (when indicated):

$$y = \alpha_0 + \alpha_1\, LBS + \alpha_2 Year + \ \alpha_3 LBS\ x\ Year + \epsilon$$

Where year is a continuous variable and trinity indicates whether the physician is in the Larger Bonus Size (LBS) group.[2] Physicians are included only if they are included in the main analysis.

This analysis demonstrated no significant differences in the trend in performance (Year x Trinity interaction term) in the years prior to the intervention.

[1] The Ischemic Vascular disease measures were for a broader set of patients in the main analysis.
[2] Year is centered at 2010 to ease interpretation of the coefficient on Trinity

© 2019 Navathe AS et al. *JAMA Network Open*.

eTable 2. Complete Unadjusted Results of Randomized Clinical Trial

Study Measure	Increased Social Pressure + Larger Bonus Size					Loss Aversion + Larger Bonus Size				
	No. of Patients	2015	No. of Patients	2016	Difference	No. of Patients	2015	No. of Patients	2016	Difference
**Overall**	**1496**	**85%**	**1496**	**89%**	**4%**	**1387**	**84%**	**1387**	**88%**	**4%**
**Asthma Action Plan**	92	86%	72	91%	5%	46	78%	42	87%	9%
**Asthma Control Treatment Assessed**	92	84%	72	91%	8%	46	78%	42	86%	8%
**Asthma Medication Management**	53	94%	35	97%	2%	19	95%	20	94%	0%
**Adult BMI**	737	96%	768	95%	-1%	622	98%	669	96%	-2%
**Blood Pressure Control (<140/90 mm/Hg)**	1388	83%	1406	84%	1%	1307	85%	1326	85%	0%
**IVD/CAD – Blood Pressure Measurement**	1228	96%	1290	96%	0%	1252	98%	1259	98%	0%
**COPD Spirometry Evaluation**	239	54%	288	65%	11%	199	72%	221	81%	9%
**Diabetes: Eye Exam Performed**	586	62%	608	68%	5%	416	55%	430	64%	9%
**Diabetes: Foot Exam**	585	74%	608	89%	15%	416	88%	430	87%	-1%
**HbA1c Control (<8%)**	586	69%	608	72%	4%	416	61%	430	66%	5%
**HbA1c Poor Control (>9%)**	586	77%	608	82%	5%	416	73%	430	76%	3%
**Hemoglobin A1c Testing**	586	96%	608	94%	-2%	416	94%	430	94%	0%
**Diabetes: Medical Attention for Nephropathy**	585	96%	608	96%	0%	416	97%	430	97%	-1%
**CHF Appropriate Medication Outpatient – ACEi or ARBs**	90	80%	64	92%	12%	88	90%	75	91%	1%
**CHF Appropriate Medication Outpatient – Beta Blockers**	26	54%	18	100%	46%	28	89%	23	93%	4%
**IVD/CAD – Use of Anti-Platelet Medication**	198	80%	220	91%	11%	242	90%	273	91%	2%
**Depression Screening and Follow Up Plan**	1233	92%	1233	99%	6%	1172	97%	1172	99%	2%
**Documentation of Designated Decision Maker for Medical Care Form**	539	37%	584	72%	36%	672	10%	682	42%	32%
**Tobacco Use Cessation Counseling**	334	87%	317	92%	5%	352	80%	269	89%	9%
**Tobacco Use Assessment**	1486	97%	1487	99%	1%	1384	98%	1384	99%	1%

Abbreviations**:** BMI, Body Mass Index; IVD, Ischemic Vascular Disease; CAD, Coronary Artery Disease; COPD, Chronic Obstructive Pulmonary Disease; HbA1c, Hemoglobin A1c; CHF, Congestive Heart Failure; ACEi, Angiotensin-converting enzyme (ACE) inhibitor; ARBs, Angiotensin II receptor blockers

Larger Bonus Size Only	Adjusted Pair-Wise Comparison[a]

© 2019 Navathe AS et al. *JAMA Network Open*.

# Patients	2015	# Patients	2016	Difference	ISP vs LA 2016 vs 2015	ISP vs AC 2016 vs 2015	LA vs AC 2016 vs 2015
864	88%	864	92%	4%			
55	95%	52	94%	-1%	>0.99	>0.99	>0.99
55	93%	52	93%	0%	>0.99	>0.99	>0.99
23	100%	21	100%	0%	>0.99	>0.99	>0.99
316	92%	359	96%	4%	>0.99	>0.99	0.73
671	84%	730	89%	5%	>0.99	>0.99	>0.99
608	95%	667	98%	3%	>0.99	0.32	0.03 [a]
248	81%	265	87%	6%	>0.99	>0.99	>0.99
231	69%	261	76%	7%	>0.99	>0.99	>0.99
231	85%	261	88%	3%	0.91	>0.99	>0.99
231	58%	261	71%	12%	>0.99	>0.99	>0.99
231	70%	261	80%	10%	>0.99	>0.99	>0.99
231	89%	261	93%	4%	>0.99	>0.99	>0.99
231	97%	261	97%	0%	>0.99	>0.99	>0.99
35	91%	49	91%	0%	>0.99	>0.99	>0.99
12	83%	13	98%	15%	0.15	>0.99	>0.99
107	93%	111	94%	1%	>0.99	>0.99	0.98
622	95%	665	99%	3%	0.80	>0.99	>0.99
296	54%	344	79%	24%	>0.99	>0.99	>0.99
163	90%	179	93%	3%	>0.99	>0.99	>0.99
845	98%	845	98%	0%	>0.99	>0.99	>0.99

[a] Reported p-values for pairwise comparisons of the primary outcome of change in proportion of applicable chronic disease and preventive evidence-based measures meeting or exceeding benchmarks at the patient level use the Holm-Bonferroni correction. Multiple imputation was used for the approximately 11% of participants missing follow-up quality metric scores.

© 2019 Navathe AS et al. *JAMA Network Open*.

eTable 3. Complete Unadjusted Results of Cohort Study

Study Measure	Larger Bonus Size					No Larger Bonus Size (comparison group)					Adjusted Pair-Wise Comparison
	No. of Patients	2015	No. of Patients	2016	Difference	No. of Patients	2015	No. of Patients	2016	Difference	Adjusted P-Value[a]
**Overall**	**3747**	**85%**	**3747**	**89%**	**4%**	**4371**	**86%**	**4371**	**88%**	**2%**	
**Asthma Action Plan**	193	87%	166	91%	4%	164	84%	128	88%	4%	>.99
**Asthma Control Treatment Assessed**	193	85%	166	90%	5%	164	81%	129	88%	7%	.95
**Asthma Medication Management**	95	96%	76	97%	1%	104	93%	74	100%	7%	>.99
**Adult BMI**	1675	96%	1796	96%	0%	2119	97%	2168	95%	-2%	.12
**Blood Pressure Control (<140/90 mm/Hg)**	3366	84%	3462	86%	2%	4086	89%	4114	84%	-4%	.00
**IVD/CAD – Blood Pressure Measurement**	3088	97%	3216	97%	0%	3820	98%	3891	97%	-1%	.16
**COPD Spirometry Evaluation**	686	69%	774	77%	8%	745	69%	855	72%	3%	.08
**Diabetes: Eye Exam Performed**	1233	61%	1299	68%	7%	1235	64%	1218	66%	2%	.16
**Diabetes: Foot Exam**	1232	81%	1299	88%	8%	1235	81%	1218	82%	0%	.00
**HbA1c Control (<8%)**	1233	64%	1299	70%	6%	1235	72%	1219	71%	0%	.08
**HbA1c Poor Control (>9%)**	1233	74%	1299	80%	5%	1235	81%	1219	81%	0%	.09
**Hemoglobin A1c Testing**	1233	94%	1299	94%	0%	1235	95%	1219	94%	-1%	>.99
**Diabetes: Medical Attention for Nephropathy**	1232	97%	1299	97%	0%	1235	96%	1219	96%	0%	>.99
**CHF Appropriate Medication Outpatient – ACEi or ARBs**	213	86%	188	91%	5%	261	86%	205	91%	5%	>.99
**CHF Appropriate Medication Outpatient – Beta Blockers**	66	74%	54	97%	22%	80	91%	75	93%	2%	.70

© 2019 Navathe AS et al. *JAMA Network Open*.

**IVD/CAD – Use of Anti-Platelet Medication**	547	87%	604	91%	5%	1061	89%	1118	91%	2%	>.99
**Depression Screening and Follow Up Plan**	3027	95%	3070	99%	4%	3565	93%	3559	98%	5%	>.99
**Documentation of Designated Decision Maker for Medical Care Form**	1507	29%	1610	61%	33%	2060	27%	2162	54%	28%	.17
**Tobacco Use Cessation Counseling**	849	85%	765	91%	6%	698	92%	669	91%	-1%	.04
**Tobacco Use Assessment**	3715	98%	3716	99%	1%	4341	99%	4343	99%	0%	>.99

[a] Reported p-values for pairwise comparisons of the primary outcome of change in proportion of applicable chronic disease and preventive evidence-based measures meeting or exceeding benchmarks at the patient level use the Holm-Bonferroni correction. Multiple imputation was used for the approximately 11% of participants missing follow-up quality metric scores. Abbreviations**:** BMI, Body Mass Index; IVD, Ischemic Vascular Disease; CAD, Coronary Artery Disease; COPD, Chronic Obstructive Pulmonary Disease; HbA1c, Hemoglobin A1c; CHF, Congestive Heart Failure; ACEi, Angiotensin-converting enzyme (ACE) inhibitor; ARBs, Angiotensin II receptor blockers

eTable 4. Sample Characteristics of Cohort Study for Larger Bonus Size Without Matching

		**Larger Bonus Size**	**All No Larger Bonus Size**	***P* Value**
**Number of physicians**		N = 33	N = 801	
**Age (year), mean (SD)**		57 (10)	53 (10)	.04
**Tenure (year), mean (SD)**		12 (8)	9 (7)	.03
**Average No. of APP patients in panel, median (IQR)**		67 (138)	34 (131)	.06
**Gender, No. (%)**				
	Female	15 (45%)	285 (36%)	.25
	Male	18 (55%)	516 (64%)	
**Specialty, No. (%)[a]**				.00

© 2019 Navathe AS et al. *JAMA Network Open*.

Family Medicine	14 (42%)	153 (19%)	
Internal Medicine	13 (39%)	214 (27%)	
Pediatrics	4 (12%)	183 (23%)	
Others	2 (6%)	251 (31%)	
**Average No. of Chronic Disease, mean (SD)**	1.60 (0.34)	1.47 (0.38)	.05
**Number of patients**	N = 3747	N = 70818	
**Age (year), median (IQR)**	64 (18)	68 (18)	<.0001
**Gender, No. (%)**			
Female	2384 (64%)	36880 (52%)	<.0001
Male	1358 (36%)	33758 (48%)	
**Race, No. (%)**			
Black or African American	2667 (71%)	7461 (11%)	<.0001
Caucasian or White	368 (10%)	48658 (69%)	
Other	149 (4%)	4547 (6%)	
Unknown	563 (15%)	10152 (14%)	
**Average No. of Chronic Disease, mean (SD)**	1.6 (0.82)	1.63 (0.83)	.06

[a]Other physicians includes 1 Cardiologist and 1 Pulmonologist in the Larger Bonus Size cohort. For No Larger Bonus Size cohort, Other physicians includes 28 Allergists/Immunologists, 5 Cardiac Electrophysiologists, 98 Cardiologists, 25 Endocrinologists, 10 Interventional Cardiologists, 6 Pediatric Allergists/Immunologists, 79 Pulmonologists

Abbreviations: SD, standard deviation; IQR, interquartile range.

© 2019 Navathe AS et al. *JAMA Network Open*.

eFigure 5. Sensitivity Analysis for RCT Without Physician Fixed Effect Clustering at Group Practice Level

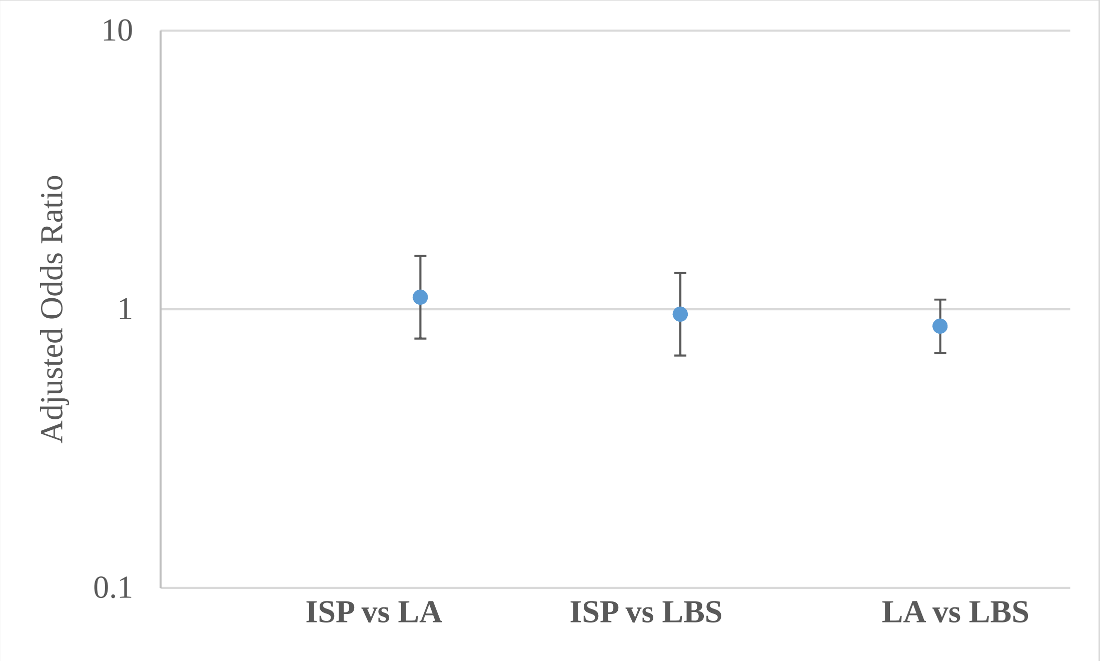

Error bars indicate 95% confidence Intervals

ISP: Larger bonus size + Increased social pressure
LA: Larger bonus size + Loss aversion
LBS: Larger bonus size only (comparison group)

© 2019 Navathe AS et al. *JAMA Network Open*.

eFigure 6. Sensitivity Analysis for RCT Without Imputation (Using Complete Case Data)

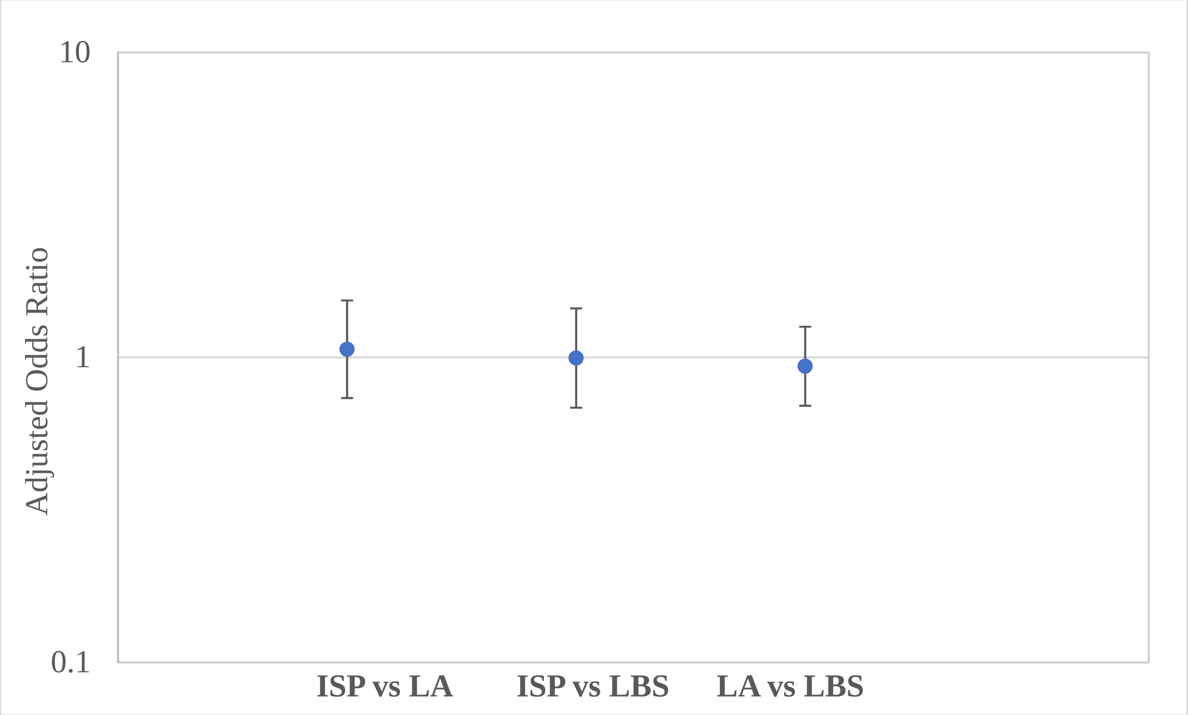

Error bars indicate 95% confidence Intervals

ISP: Larger bonus size + Increased social pressure
LA: Larger bonus size + Loss aversion
LBS: Larger bonus size only (comparison group)

© 2019 Navathe AS et al. *JAMA Network Open*.

eFigure 7. Sensitivity Analysis for RCT With Physician Random Effect

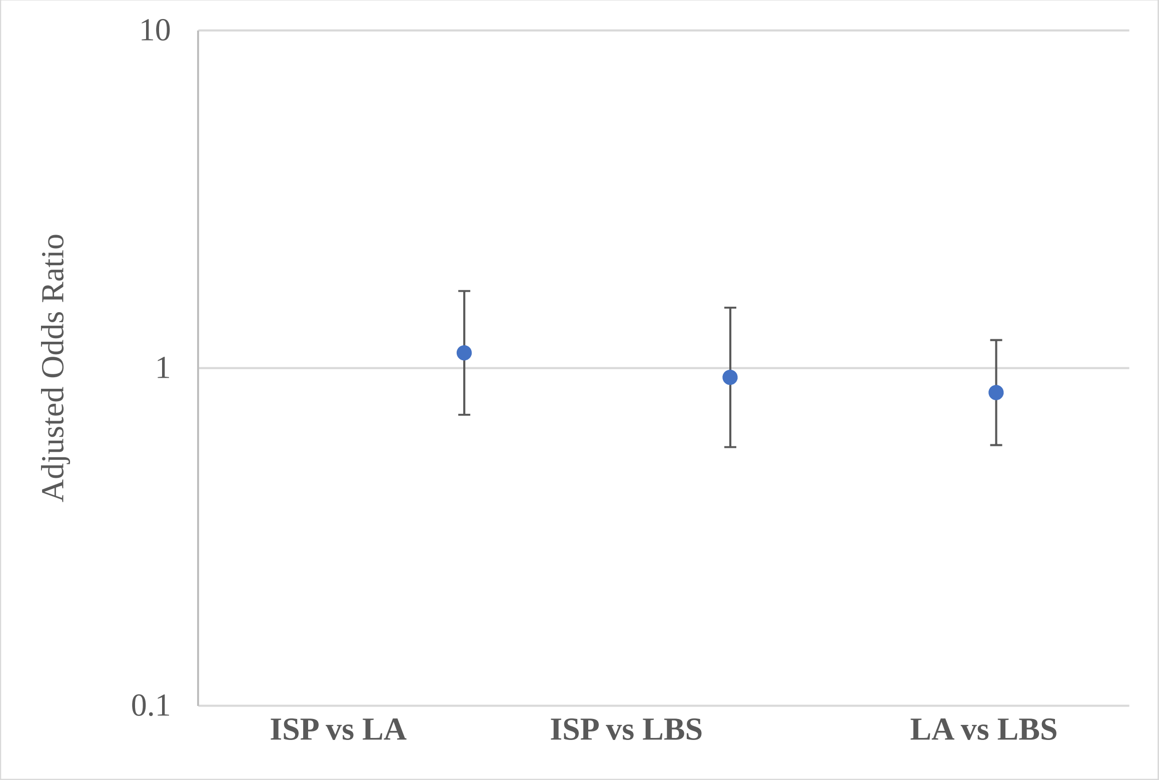

Error bars indicate 95% confidence Intervals

ISP: Larger bonus size + Increased social pressure
LA: Larger bonus size + Loss aversion
LBS: Larger bonus size only (comparison group)

© 2019 Navathe AS et al. *JAMA Network Open*.

eFigure 8. Sensitivity Analysis of Cohort Study Without Imputation (Using Complete Case Data)

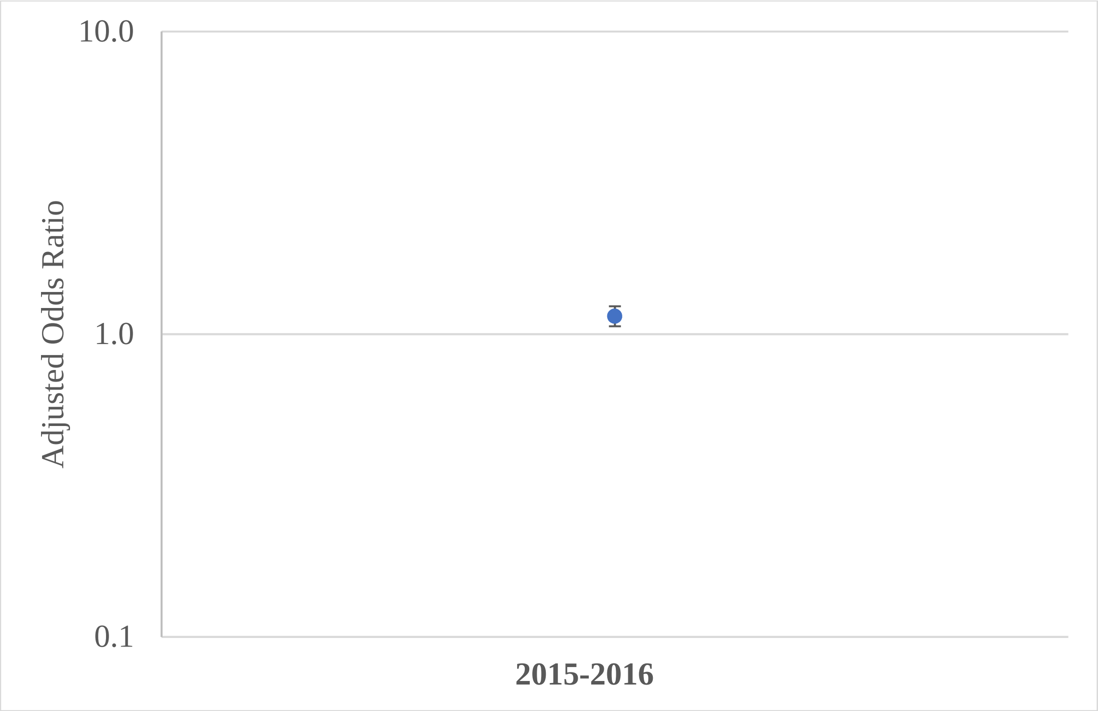

The estimate is the effect of the association between larger bonus size and higher achievement of evidence-based quality measures. The error bars indicate 95% confidence intervals.

© 2019 Navathe AS et al. *JAMA Network Open*.

eFigure 9. Sensitivity Analysis of Cohort Study Without Physician Fixed Effects

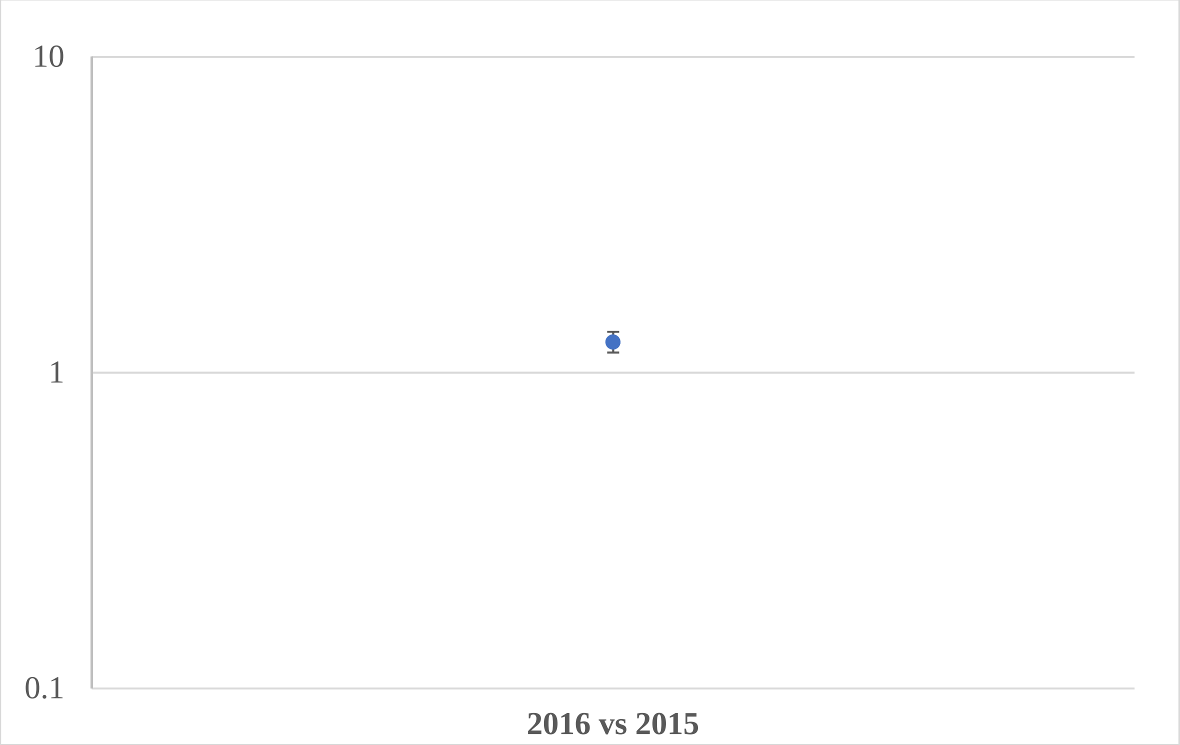

The estimate is the effect of the association between larger bonus size and higher achievement of evidence-based quality measures. The error bars indicate 95% confidence intervals.

© 2019 Navathe AS et al. *JAMA Network Open*.

eTable 5. Test of Trends for Difference-in-Differences Model Results

Coefficient (SE)	All Physicians, Weighted	Stable Set of Physicians, Weighted
Year	-0.007	-0.006
	(0.005)	(0.004)
Trinity	-0.013	-0.009
	(0.031)	(0.030)
Year x Trinity	-0.011	-0.012
	(0.008)	(0.007)
Constant	0.854***	0.851***
	(0.020)	(0.019)
Observations	186	165
R^2	0.116	0.112
Unique Trinity MDs	32	18
Unique Non-Trinity MDs	33	23

Standard errors in parentheses; * $p < 0.05$, ** $p < 0.01$, *** $p < 0.001$

© 2019 Navathe AS et al. *JAMA Network Open*.

eTable 6. Results of Physician Survey Administered Before and After Intervention

Overall	Larger Bonus Size				Loss Aversion & Larger Bonus Size				Increased Social Pressure & Larger Bonus Size			
	**Pre**	**Post**	**Change**	**t-test**	**Pre**	**Post**	**Change**	**t-test**	**Pre**	**Post**	**Change**	**t-test**
	**n=24**	**n=14**			**n=26**	**n=13**			**n=21**	**n=7**		
**Baseline Attitudes**	4.21	4.18	-0.04	0.47	3.64	3.69	0.06	0.45	3.98	4.02	0.04	0.44
**Teamwork**	3.89	3.91	0.03	0.48	4.11	3.93	-0.18	0.30	4.18	3.82	-0.37	0.02
**Financial Salience**	3.61	3.36	-0.25	0.33	3.03	3.69	0.67	0.04	3.35	3.35	0.01	0.41
**Practice Environment**	3.69	3.57	-0.12	0.37	4.00	3.80	-0.20	0.04	3.35	3.35	0.01	0.41
**Awareness/Understanding**	3.54	3.77	0.23	0.32	3.67	3.67	0.00	0.50	3.40	3.37	-0.03	0.45
**Individual Impact on Clinical Behavior**	3.48	3.57	0.10	0.43	3.37	3.22	-0.15	0.26	3.47	3.46	-0.01	0.48
**Unintended Consequences**	2.83	3.10	0.27	0.14	2.85	3.33	0.48	0.01	3.14	3.25	0.11	0.25

© 2019 Navathe AS et al. *JAMA Network Open*.
