# Supplementary material for: Effect of Financial Bonus Size, Loss Aversion, and Increased Social Pressure on Physician Pay-for-Performance: A Randomized Clinical Trial and Cohort Study
Source: JAMA Netw Open. 2019 Feb 8;2(2):e187950. doi: 10.1001/jamanetworkopen.2018.7950 (PMC6484616; doi:10.1001/jamanetworkopen.2018.7950)
Supplement: Supplement 3. — Data Sharing Statement [file jamanetwopen-2-e187950-s003.pdf]

Data Sharing Statement

Navathe. Effect of Financial Bonus Size, Loss Aversion, and Increased Social Pressure on Physician Pay-for-Performance. *JAMA Netw Open*. Published February 08, 2019. 10.1001/jamanetworkopen.2018.7950

Data
Data available: No
